# Unimolecular dual-reporter probes for early kidney injury diagnosis through synchronous *in situ* imaging and portable urinalysis

**DOI:** 10.1093/nsr/nwag367

**Published:** 2026-06-12

**Authors:** Lingyan Liu, Feiyang Liu, Qihang Ding, Shasha Wang, Peng Wei, Jong Seung Kim, Tao Yi

**Affiliations:** State Key Laboratory of Advanced Fiber Materials, College of Chemistry and Chemical Engineering, Donghua University, Shanghai 201620, China; Guangxi Key Laboratory of Special Biomedicine, School of Medicine, Guangxi University, Nanning 530004, China; Department of Chemistry, Korea University, Seoul 02841, Korea; State Key Laboratory of Advanced Fiber Materials, College of Chemistry and Chemical Engineering, Donghua University, Shanghai 201620, China; State Key Laboratory of Advanced Fiber Materials, College of Chemistry and Chemical Engineering, Donghua University, Shanghai 201620, China; Department of Chemistry, Korea University, Seoul 02841, Korea; State Key Laboratory of Advanced Fiber Materials, College of Chemistry and Chemical Engineering, Donghua University, Shanghai 201620, China

**Keywords:** unimolecular dual-reporter, early diagnosis, acute kidney injury, multiplexed signal output, portable urinalysis

## Abstract

Early detection of acute kidney injury (AKI) is critical for effective intervention and improved survival, yet it remains challenging due to the lack of practical tools that sensitively capture subtle renal dysfunction. Here, we report a unimolecular dual-reporter probe, **DHU-AKI-3**, featuring activatable crosstalk-free multiplexed signal output for precise diagnosis of early AKI by integrating *in situ* imaging and *in vitro* portable urinalysis. Following systemic administration, **DHU-AKI-3** is selectively activated in the injured kidney, producing a long-retained photoacoustic signal for real-time *in situ* imaging ahead of the clinical diagnostic window. Concurrently, a near-infrared fluorescent signal is rapidly released and excreted into urine, enabling smartphone-assisted colorimetric analysis for noninvasive, point-of-care testing. The mutual validation of crosstalk-free *in situ* and *in vitro* signals guaranteed the diagnostic accuracy. This study underscores the potential of unimolecular dual-reporter probe for directly visualizing early-stage AKI in urine, meeting the pressing demand for point-of-care testing in AKI management.

## Teaser

A unimolecular dual-reporter probe integrates in situ imaging and portable urinalysis, enabling accurate and noninvasive diagnosis of early kidney injury.

## INTRODUCTION

Acute kidney injury (AKI) is a common and life-threatening condition, marked by a sharp loss of renal function. It affects ∼15% of hospitalized patients and over 50% of those in intensive care units (ICUs), with high morbidity and mortality [1,2]. Since the standard biomarkers of AKI, blood urea nitrogen (BUN) and serum creatinine (SCr), do not exhibit noticeable changes until the glomerular filtration rate (GFR) has decreased by at least 50%, current diagnostic approaches are accurate and effective only in the advanced stage [3,4]. The renal function has already suffered severe and irreversible damage, potentially progressing to kidney failure by the time AKI is diagnosed clinically. Studies have shown that kidney failure accounts for ∼2 million deaths annually [5]. Thus, effective diagnosis of early AKI is crucial for timely therapeutic intervention and alleviating the risk of mortality.

**Figure 1.**
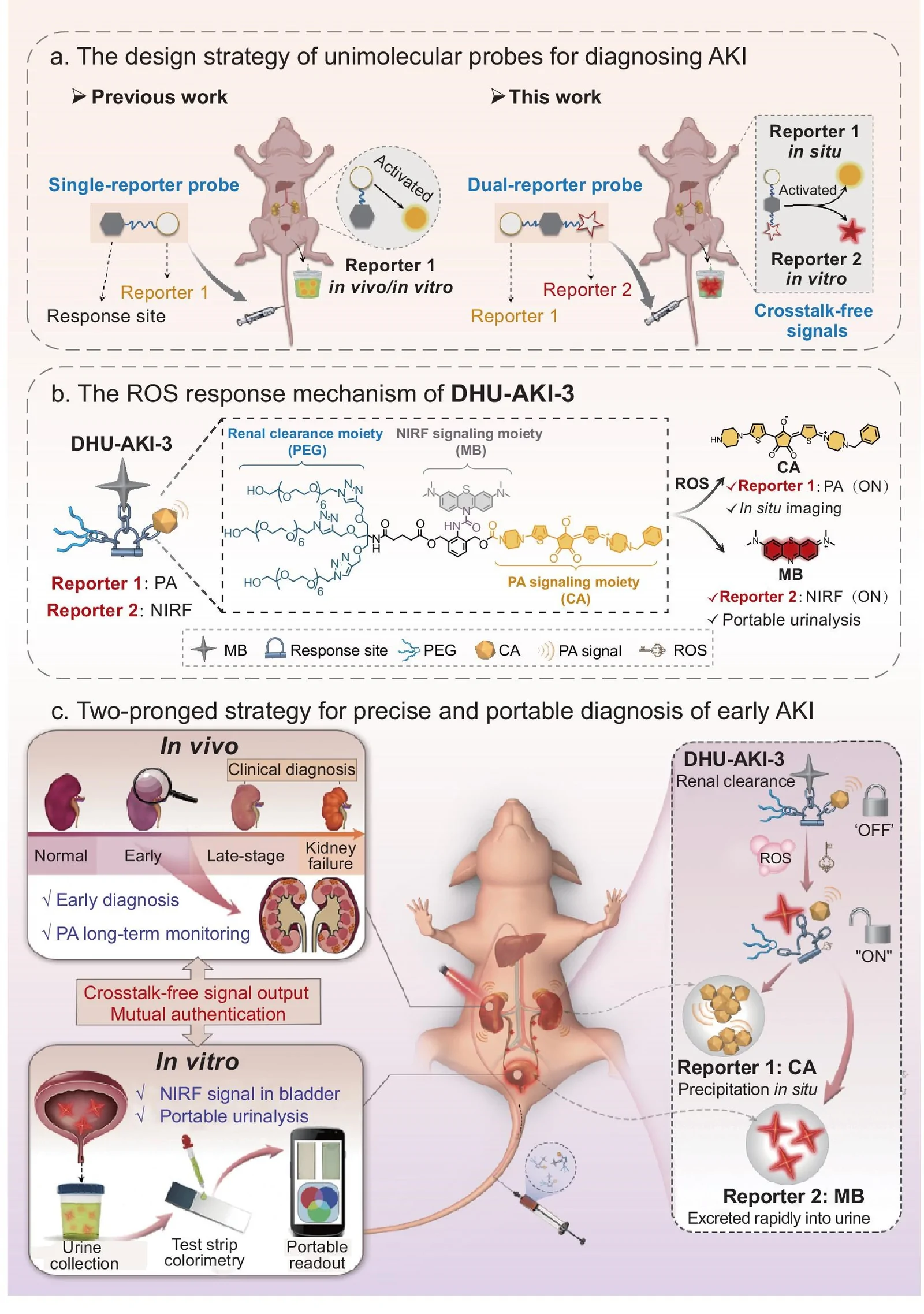
Design and mechanisms of unimolecular dual-reporter probes with crosstalk-free multiplexed signal output for precise diagnosis of early AKI. (a) The design strategy of unimolecular probes for diagnosing AKI. (b) The chemical structure of **DHU-AKI-3** and its released two reporters activated by ROS. (c) Schematic illustration showing the integration of *in situ* and *in vitro* detection of **DHU-AKI-3** by crosstalk-free multiplexed output for precise diagnosis of early kidney injury in a living mouse.

Noninvasive imaging techniques such as positron emission tomography (PET), computed tomography (CT), and magnetic resonance imaging (MRI) have been widely employed to assess renal function by monitoring the clearance of contrast agents [6–8]. However, their widespread applications are limited by equipment inaccessibility, insufficient spatiotemporal resolution, nonspecific accumulation of ‘always-on’ signals, and potential radiation risk [7]. In contrast, activatable optical probes offer high sensitivity, rapid response, and modular design, making them attractive for molecular sensing and disease diagnosis [9–12]. Yet a key point to their rational design lies in identifying suitable activators. Specifically, reactive oxygen species (ROS), established as early indicators of AKI due to their abnormal fluctuations are more sensitive than proteins [13–16], serve as sensitive activators that have spurred the development of various ROS-activated optical probes for AKI diagnosis (see Table S1 in the Supporting Information (SI)) [17–23]. However, most ROS-activated probes developed for AKI diagnosis employ a single reporter for either *in vivo* optical imaging or *in vitro* urinalysis, lacking the capability for synergistic diagnosis (Fig. 1a). On the one hand, the single-reporter-based diagnostic strategy is vulnerable to interference from frequent urination, leading to poor *in vivo* diagnostic precision [24]. On the other hand, the diagnostic specificity of urinalysis remains limited due to the lack of direct correlation between urine signals and the kidney’s actual damage *in situ* [25]. Therefore, a two-pronged strategy based on a unimolecular dual-reporter design simultaneously combining *in vivo* monitoring and *in vitro* urinalysis is anticipated to greatly improve the accuracy and timeliness of early AKI detection. However, owing to the challenges of achieving stable signals under conditions of frequent urination and the difficulties in constructing multifunctional probes with crosstalk-free multiplexed output, the two-pronged strategy has remained unrealized.

To overcome these challenges, we developed a unimolecular dual-reporter platform that enables crosstalk-free multiplexed signal output to allow for *in situ* long-term imaging and portable urinalysis of early AKI (Fig. 1a). Each probe is tailored by three key blocks: photoacoustic (PA) indicator block (reporter 1: croconic acid (CA), absorption bands at ∼800 nm) [26–28], ROS-activated near-infrared fluorescence (NIRF) block (reporter 2: methylene blue (MB)) [29,30], and hydrophilic block (polyethylene glycol, PEG), which is essential for renal clearance (Scheme S1) [31]. The representative probe **DHU-AKI-3** exhibits a PA signal (CA, reporter 1) and can be activated rapidly by ROS to release the ‘turn-on’ NIRF signal (MB, reporter 2) because of the cleavage of the urea bond by ROS (Fig. 1b). Owing to its high hydrophilicity, **DHU-AKI-3** is predominantly filtered through the glomerulus and reabsorbed by renal tubules, ensuring preferential accumulation in kidneys. Following systemic administration, **DHU-AKI-3** is metabolized primarily by renal clearance and activated by ROS in injured kidneys, swiftly releasing the CA indicator and the blue-colored MB fluorophore (Fig. 1c). Subsequently, the water-soluble MB (reporter 2) is rapidly excreted into urine, enabling portable urinalysis *in vitro*, whereas the hydrophobic CA (reporter 1) precipitates in the damaged kidney for *in situ* PA imaging. The precipitation-induced retention effect prolongs the intrarenal signal duration while facilitating the validation of portable urinalysis results [32]. This unimolecular dual-reporter two-pronged strategy provides an accurate and portable approach for early diagnosis of AKI, highlighting its potential for real-time monitoring of AKI progression at the point of care.

## RESULTS AND DISCUSSION

### Design and synthesis

To design a unimolecular dual-reporter probe that enables crosstalk-free multiplexed signal output, three specific attributes are considered. (1) It exhibits good water solubility in the intrinsic state, enabling its accumulation in the kidney. (2) It can be quickly activated by ROS to produce an accumulated signal that will not be cleared easily in a short time, thereby reducing the influence of frequent urination. (3) It releases a multiplexed signal with completely distinct metabolic behaviors, avoiding crosstalk between the same type of signal output to ensure signal retention in the kidneys and output in urine. Based on these considerations, we performed a retrosynthesis analysis before the synthesis of the probes **DHU-AKI-1, 2, 3** (see Schemes S1 and S2). For example, **DHU-AKI-3** included three main moieties: a PEG cluster, a CA chromophore, and a ROS-responsive fragment **7** that contained leucomethylene blue (LMB) and (2-amino-1,3-phenylene) dimethanol conjugated through a urea bond. The PEG cluster and CA were symmetrically linked to the benzyl alcohol of the ROS-responsive fragment. First, we synthesized CA as a main block in **DHU-AKI-3** using compound **1** as the starting material and introduced a benzyl group using a classic substitution reaction to increase its hydrophobicity (Scheme S3). We obtained the highly ROS-sensitive key precursor **7** with multiple modifiable sites (benzyl alcohol) from our previous work [29,30]. To improve the reaction yield, **7** was symmetrically protected by silicon to give compound **8**. Subsequently, a substitution reaction took place between the amino group of the CA dye and unprotected benzyl alcohol of **8** after it was activated with 4-nitrophenyl chloroformate to offer **15**, in which the silane-protected group was subsequently removed to provide **DHU-AKI-1** (Scheme S4). Subsequently, to improve the kidney-targeting performance, probes were conjugated to hydrophilic PEG with varying molecular weights, yielding **DHU-AKI-2** and **DHU-AKI-3. DHU-AKI-4** without CA was also synthesized as a reference compound (Scheme S5). The intermediates and final compounds were characterized by nuclear magnetic resonance (NMR) spectroscopy and high-resolution mass spectrometry (HR-MS) (Figs S20–S38).

### *In vitro* characterization

**Figure 2.**
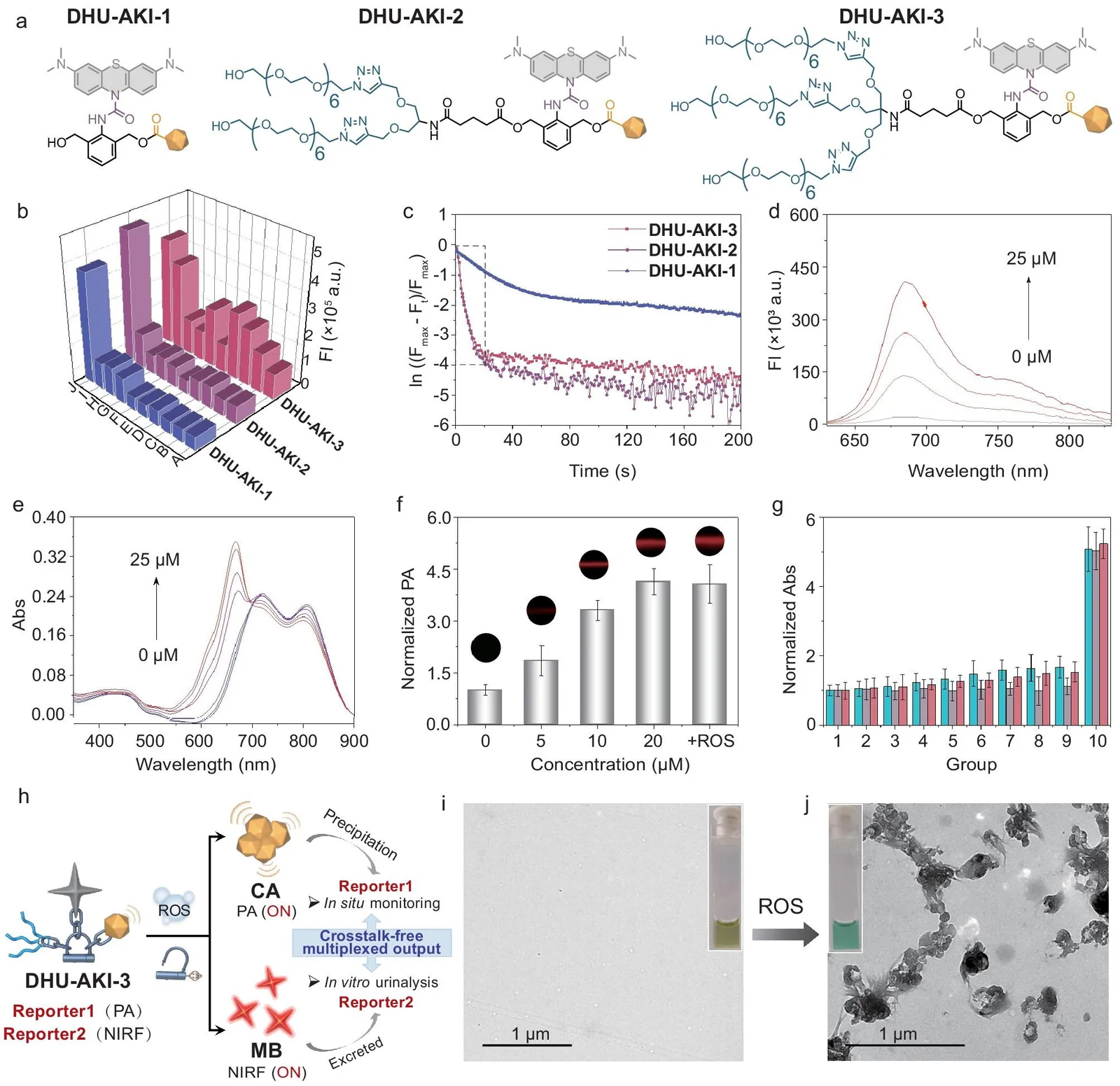
Evaluation of the optical properties and response capability of probes *in vitro*. (a) The chemical structure of probes. (b) The fluorescence intensity (FI) of reporters (10 μM in PB) at 686 nm after addition of various ROS, A–J: only reporter, H_2_O_2_ (80 μM), O_2_^•−^ (80 μM), NO (80 μM), ROO^•^ (80 μM), TBHP (80 μM), ^•^OH (80 μM), *t*-BuOO^•^ (80 μM), ONOO^−^ (80 μM), and HOCl (40 μM). (c) Pseudo-first-order kinetic plot of reporters (10 μM) in the presence of equilibrium concentrations of HOCl. (d) The changes in fluorescence spectra of 10 μM **DHU-AKI-3** before and after adding various concentrations of HOCl in PB (pH 7.4). (e) The changes in absorption (Abs) spectra of **DHU-AKI-3** (10 μM in PB) upon adding different concentrations of HOCl. (f) PA intensity of **DHU-AKI-3** with different concentrations (0, 5, 10 and 20 μM in PB) and 20 μM **DHU-AKI-3** after incubation with HOCl (20 μM, +ROS group represents 20 μM HOCl) at 805 nm. Data are the mean ± s.d. for *n* = 3. (g) Stability test: the absorption intensity changes of **DHU-AKI-3** (10 μM in PB) at 664 nm in different solutions of the time series (Cyan: MEM; Brown: 10% FBS; Pink: pH 7.4 PBS; 1–9: 0, 1, 2, 4, 6, 12, 24, 36, and 48 h; 10: after incubation with 25 μM HOCl. These data are the mean ± s.d.; *n =* 3). (h) Schematic illustration showing the molecular mechanism of **DHU-AKI-3** through ROS activation. (i and j) TEM images of (i) **DHU-AKI-3** (50 μM) without the addition of HOCl, (j) **DHU-AKI-3** (50 μM) in the presence of HOCl (50 μM), and the insets show the color changes (TEM images were acquired at different magnifications to optimally visualize structural features; scale bars are indicated individually. Scale bars = 1 μm).

We investigated the optical properties and response capabilities of the prepared probes by means of ultraviolet-visible (UV-vis) absorption, fluorescence spectroscopy, and PA methods in phosphate buffer (PB, pH 7.4, Fig. 2a). The ROS selectivity of various probes was initially evaluated by studying the fluorescence changes in reporters in the presence of various ROS (^•^OH, ONOO^−^, *t*-BuOO^•^, H_2_O_2_, TBHP, NO, O_2_^•−^, ROO^•^, and HOCl). As shown in Fig. 2b and Fig. S1, both **DHU-AKI-1** and **DHU-AKI-2** showed significantly enhanced fluorescence intensity at 686 nm only in the presence of hypochlorous acid (HOCl), and the fluorescence intensity gradually increased with increasing HOCl concentration. **DHU-AKI-3**, however, gave an enhanced fluorescence signal after a reaction with multiple ROS (ONOO^−^, H_2_O_2_, TBHP, NO, O_2_^•−^, and HOCl). The broad-spectrum ROS-activatability of **DHU-AKI-3** improved its bioavailability for application in complex organisms. Using HOCl as the representative activator, the time-dependent response curves of these probes were tested. As shown in Fig. S2, the fluorescence intensity at 686 nm of **DHU-AKI-2** and **DHU-AKI-3** rapidly increased to a plateau within ∼10 s upon the addition of HOCl, which was far faster than that of **DHU-AKI-1** (∼60 s). This rapid response was beneficial to detect ROS with a short half-life in living systems. Under pseudo-first-order conditions, the rate constants were 0.0349, 0.1942, and 0.1867 s^−1^ for **DHU-AKI-1, DHU-AKI-2**, and **DHU-AKI-3**, respectively (Fig. 2c). In addition, the calculated logP values of **DHU-AKI-1, 2**, and **3** are 7.03, 1.39, and − 1.43, respectively (see Table S2 in SI). These results demonstrated that **DHU-AKI-3** exhibits good water solubility and is expected to promote its accumulation in the kidney. Therefore, considering the broad-spectrum ROS activation, rapid response, and excellent water solubility properties, we selected **DHU-AKI-3** as the probe candidate for the following study.

The fluorescence, absorption, and PA spectra in PB solution were measured to evaluate the properties of **DHU-AKI-3**. As shown in Fig. 2d, **DHU-AKI-3** showed a negligible fluorescence signal in the absence of ROS. Upon the addition of various concentrations of HOCl; however, the maximum fluorescence emission at ∼686 nm increased in a concentration-dependent manner. The detection limit of the **DHU-AKI-3** response to HOCl was 28.5 μM based on the 3*σ*/*k* method (Fig. S3). In addition, **DHU-AKI-3** showed double absorption peaks of CA at 718 and 805 nm, but a new absorption peak at ∼664 nm appeared with the addition of HOCl, which indicates the release of the MB fluorophore (Fig. 2e). The release efficiency of MB was measured to be ∼49% (Fig. S4). Meanwhile, we observed a gradually increasing PA signal at 805 nm with the increasing concentration of **DHU-AKI-3**, and the PA intensity remained consistent before and after responding with HOCl (Fig. 2f). This property was favorable for real-time tracking of **DHU-AKI-3**  *in vivo*. To further evaluate the potential of **DHU-AKI-3** for application *in vivo*, we performed anti-interference experiments by incubating **DHU-AKI-3** with various physiologically relevant cations, anions and amino acids. The results showed that these species caused negligible interference with the optical response of the probe, confirming its strong resistance to biological interference under physiological conditions (Fig. S5). Even under high concentrations of various reductive species (glutathione, N-Acetyl-L-cysteine, glucose and formaldehyde), the probe still retained a reliable response (Fig. S6). The probe’s response performance under different pH conditions indicated that **DHU-AKI-3** exhibited good intrinsic stability in varying pH solutions, and could produce significantly enhanced fluorescence at 686 nm over a physiological pH range (4–8) after the addition of HOCl (Fig. S7). The result further confirms the reliability of the probe under physiologically relevant conditions. Furthermore, we evaluated its stability by detecting its absorbance at 664 nm in different medium including phosphate buffer saline (PBS, pH 7.4), 10% fetal bovine serum (FBS), and FBS-free culture medium (MEM). As illustrated in Fig. 2g, **DHU-AKI-3** alone displayed negligible absorption in those mediums even up to 48 h, indicating its good stability for biological applications. However, the addition of HOCl induced a pronounced absorption enhancement at 664 nm. These results demonstrated the desirable properties of **DHU-AKI-3** in ROS response and crosstalk-free multiplexed signal output (Fig. 2h).

### Release of precipitated CA indicator

To verify the precipitation strategy of ROS stimulated CA release, we performed HR-MS, transmission electron microscopy (TEM), and dynamic light scattering (DLS) analysis in an aqueous solution. The free **DHU-AKI-3** could completely dissolve in water, benefiting from the modification of the PEG moiety (Fig. 2i and Fig. S8), which facilitated CA delivery to the kidneys and significantly enhanced its bioavailability *in vivo*. After **DHU-AKI-3** reacted with ROS, HR-MS data indicated the formation of MB (*m*/*z* [M + H]^+^ calcd for C_16_H_18_N_3_S^+^: 284.1216, found: 284.1195) and CA (*m*/*z* [M + H]^+^ calcd for C_28_H_28_N_4_O_3_S_2_ : 533.1681, found: 533.1671) (Fig. S9). As expected, we observed the larger aggregates of CA upon ROS activation of **DHU-AKI-3**, and the hydrodynamic diameter significantly increased to 0.3–4 μm (Fig. 2j and Fig. S8). Furthermore, the threshold concentration of **DHU-AKI-3** required for CA precipitation was systematically examined. As shown in Fig. S10, noticeable precipitation was observed when the concentration of **DHU-AKI-3** was 20 μM, demonstrating the rapid signal accumulation capability of **DHU-AKI-3** within a short timescale. These results confirm that **DHU-AKI-3** is capable of preventing the rapid renal clearance of *in situ* signals, highlighting its promise for long-term monitoring of kidney injury *in situ*.

### Recognition of renal tubular epithelial cell apoptosis

After confirming the ideal performance of **DHU-AKI-3**  *in vitro*, we investigated its capacity to diagnose injured renal cells using the human kidney proximal tubular epithelial cell line (HK-2) as the cell model. Initially, we evaluated the cytotoxicity of **DHU-AKI-3** through the cell counting kit-8 assay. The results showed that the survival rate of HK-2 cells remained at approximately 100% even after incubating with 300 μM **DHU-AKI-3** for 24 h, indicating that **DHU-AKI-3** is nontoxic for HK-2 cells and thus suitable for biological applications (Fig. S11). Next, the cellular uptake of **DHU-AKI-3** by HK-2 cells was investigated via confocal laser scanning microscope (CLSM) imaging. As shown in Fig. S12, CLSM images displayed no fluorescence signal in normal HK-2 cells after incubating with **DHU-AKI-3** (10 μM) for 3 h. Upon treatment with HOCl (50 μM) for different times (15, 30, and 60 min), we observed a statistically significant increase in fluorescence signal, which was observed mainly in the cytoplasm, demonstrating that **DHU-AKI-3** could be effectively internalized into HK-2 cells.

**Figure 3.**
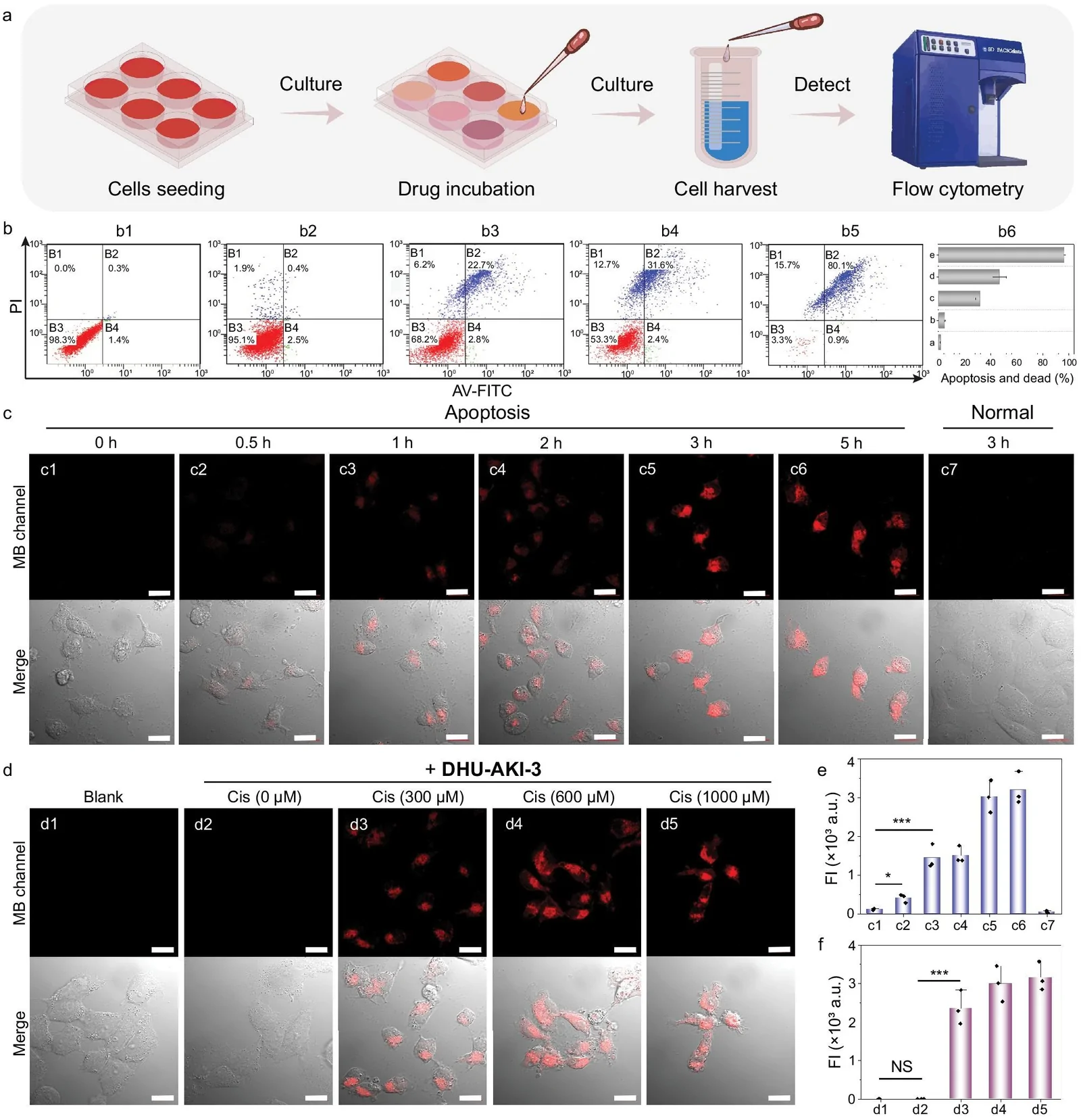
Imaging of cisplatin-induced apoptosis of HK-2 cells. (a) Schematic diagram of a cellular experiment. (b) Flow cytometer analysis of HK-2 apoptosis using Annexin V-FITC apoptosis detection kit (b1: cells without any treatment; b2: cells stained with Annexin V-FITC/PI; b3–b5: cells treated with 300 μM, 600 μM, and 1000 μM cisplatin for 12 h, respectively, before staining with Annexin V-FITC/PI; b6: the percentage of apoptosis and dead cells in b1–b5, these data are the mean ± s.d.; *n =* 3). (c) CLSM imaging of apoptosis (treated with 300 μM cisplatin) and normal HK-2 cells after incubation of **DHU-AKI-3** (10 μM) for different times. (d) CLSM imaging of HK-2 cells at different apoptosis levels (treated with 0 μM, 300 μM, 600 μM, and 1000 μM cisplatin) after incubation of **DHU-AKI-3** (10 μM) for 3 h. (e) and (f) were the average fluorescence intensity in (c) and (d), respectively. Scale bars = 20 μm, *λ*_ex_ = 633 nm. Values are the mean ± s.d. for *n =* 3, *p < 0.05, ***p < 0.001, NS: not significant.

Considering that nephrotoxic drugs such as cisplatin are the dominant factors of kidney injury [33,34], we investigated whether **DHU-AKI-3** could recognize drug-induced renal cell apoptosis. We treated HK-2 cells with different doses of cisplatin for 12 h and determined the apoptosis rate by flow cytometry using an Annexin V-FITC kit (Fig. 3a). As shown in Fig. 3b, the apoptosis rate of the HK-2 cells gradually increased with the increasing concentration of cisplatin (0, 300, 600, 1000 μM), which reached up to 81% apoptosis compared with 1.7% in normal HK-2 cells. We further studied the performance of **DHU-AKI-3** to recognize apoptotic HK-2 using CLSM imaging. For normal HK-2 cells, no fluorescence signal was observed after incubating **DHU-AKI-3** for 3 h (Fig. 3c and e). By contrast, apoptotic cells showed a gradually increased fluorescence signal with prolonged incubation time of **DHU-AKI-3** (0, 0.5, 1, 2, 3, 5 h), reaching a plateau at 3 h. In addition, cells with different degrees of apoptosis showed obviously enhanced intracellular fluorescence signals after incubation of **DHU-AKI-3** for 3 h (Fig. 3d and f). These results indicated that **DHU-AKI-3** could effectively recognize apoptotic renal tubular epithelial cells. We further verified the CLSM imaging results by TEM images of apoptotic HK-2 cells (Fig. S13). Compared with the control cells treated with FBS-free medium, the **DHU-AKI-3**-treated cells showed precipitated CA dye in the cytoplasm, demonstrating that the MB fluorophore and CA indicator were released simultaneously. These results robustly verified that **DHU-AKI-3** is a promising tool for two-pronged diagnosis of early AKI by integration of *in situ* and *in vitro* detection based on multiplexed signal output.

### Biodistribution and biosecurity evaluation

**Figure 4.**
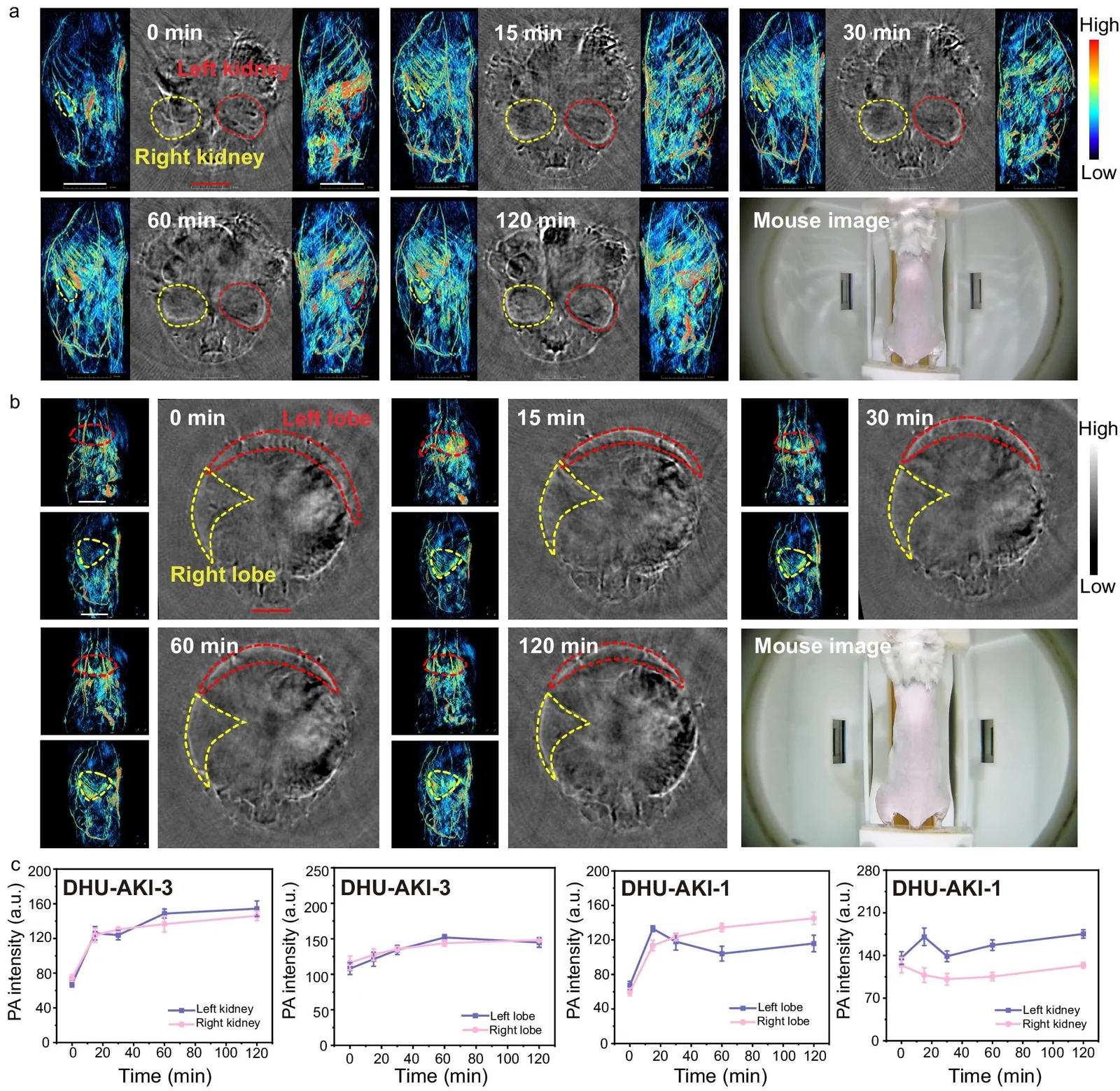
3D-PA imaging of probes *in vivo*. (a) PA imaging of AKI mice after being treated with **DHU-AKI-3** (10 mg/kg, 200 μL) at 0, 15, 30, 60, and 120 min post-injection. White scale bar: 10 mm, red scale bar: 5 mm. (b) PA imaging of AKI mice after being treated with **DHU-AKI-1** (10 mg/kg, 200 μL) at 0, 15, 30, 60, and 120 min post-injection. White scale bars: 10 mm, red scale bars: 5 mm. (c) The PA intensity of mice’s kidneys and liver in (a) and (b), values are the mean ± s.d. for *n =* 3.

For further applications *in vivo*, we investigated the biodistribution and biosecurity of **DHU-AKI-3**. We constructed a cisplatin-induced AKI mouse model according to our previous report [35], and performed the fluorescence imaging of major organs in healthy and AKI mice after intravenous administration. At 45 min postinjection, the heart, liver, spleen, lung, kidney, and muscle were harvested for *ex vivo* imaging. For healthy mice, we did not observe any significant differences in fluorescence intensity after intravenous (i.v.) injection of saline and **DHU-AKI-3**, respectively (Fig. S14a and b). For AKI mice, however, we detected a significantly increased fluorescence intensity in the kidneys only after i.v. injection of **DHU-AKI-3**, compared with the saline group (Fig. S14c–e). To more intuitively visualize the biodistribution of the probes, 3D-PA imaging was further performed to compare the real-time distribution of **DHU-AKI-3** and the control compound **DHU-AKI-1** (lacking the PEG chain) in AKI mice. As shown in Fig. 4a and c, **DHU-AKI-3** exhibited a significant PA signal enhancement in the renal region at 30 min post-injection, while the PA signal in the liver was barely detectable. Movies S1 and S2 recorded the real-time 3D-PA imaging of the above process, clearly presenting the spatial distribution of **DHU-AKI-3**. In contrast, strong PA signal accumulation of **DHU-AKI-1** was observed in the left and right lobes of the liver, while the kidney exhibited negligible signal enhancement (Fig. 4b, c, and Movies S3, S4). It indicated that lipophilic **DHU-AKI-1** was prone to being captured by the reticuloendothelial system (RES) [36,37]. These imaging results indicated that **DHU-AKI-3** was accumulated and metabolized primarily in kidneys, which is due to its excellent water solubility.

Accordingly, we further performed the blood routine analysis to evaluate the biosecurity of **DHU-AKI-3**. The major biochemical indicators of healthy mice after i.v. injection of **DHU-AKI-3** for 24 and 48 h showed no significant changes compared with the control mice (Fig. S15). In addition, we did not observe any adverse effects on renal function after administration of **DHU-AKI-3** for 24 h (Fig. S16). Histological staining of major organ tissues in the saline and **DHU-AKI-3** groups showed no obvious tissue damage or lesions in the pathological sections of those mice (Fig. S17). These results demonstrated that **DHU-AKI-3** possessed good biocompatibility and could be safely applied *in vivo*.

### *In situ* long-term monitoring of early AKI

**Figure 5.**
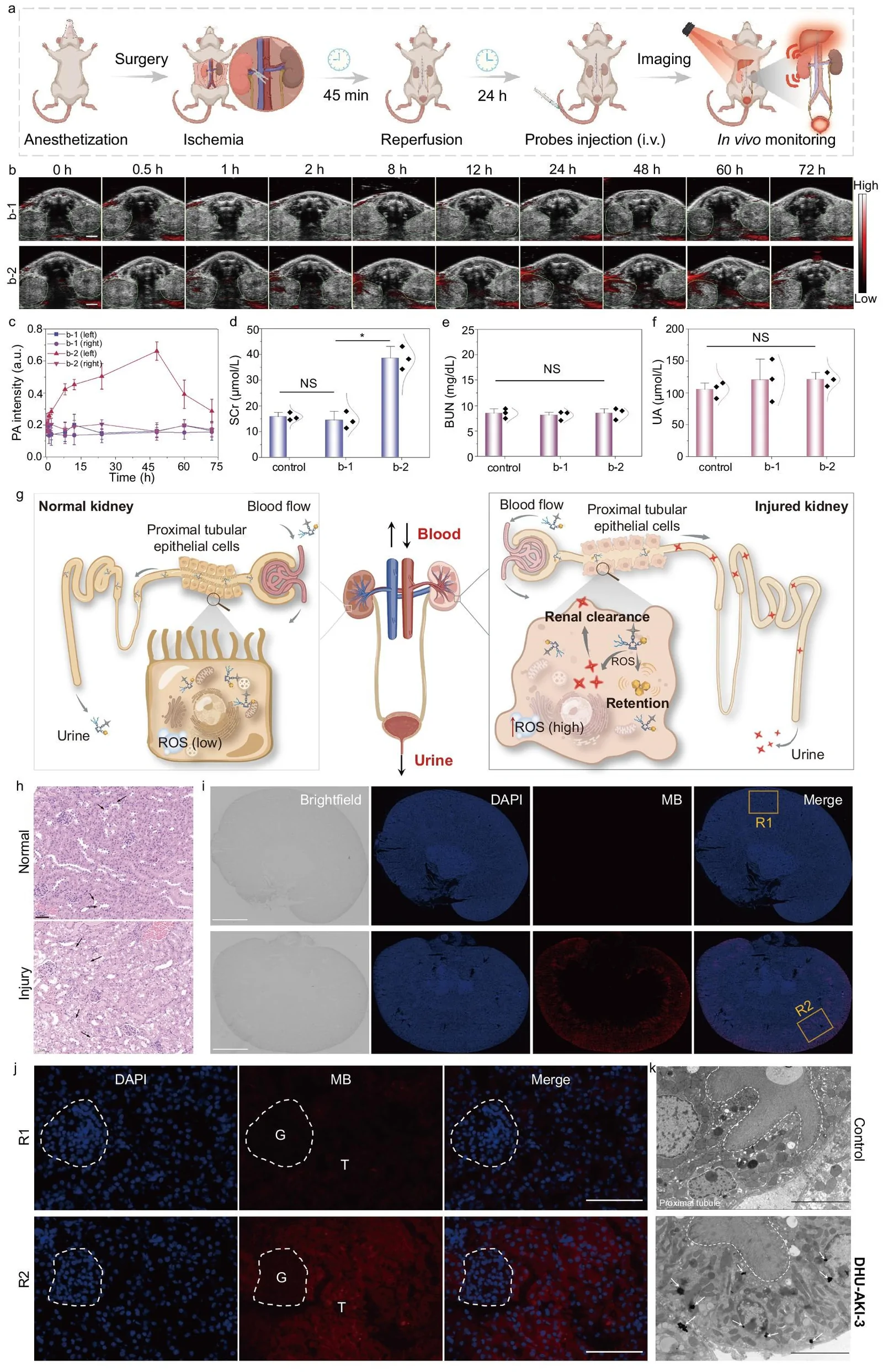
Long-term monitoring of kidney injury *in situ* and the metabolic pathways of **DHU-AKI-3** in the kidneys. (a) Schematic illustration of reporters for long-term *in-situ* monitoring of unilateral kidney (left) ischemia reperfusion injury (*n =* 3). (b) Representative PA images of mice after i.v. injection with the **DHU-AKI-3** (10 mg/kg, 200 μL) at different time points (0, 0.5, 1, 2, 8, 12, 24, 48, 60, and 72 h), scale bars = 2 mm. (c) The dynamic PA intensity of the mice’s kidneys in (b). (d–f) SCr (d), BUN (e) and UA (f) analysis of the mice in different groups of (b), *p < 0.05, NS: not significant, *n =* 3. (g) Schematic illustration of renal metabolism of **DHU-AKI-3** in the normal and injured kidney. (h) H&E staining images of the kidney from normal and AKI mice, scale bar = 50 μm. (i) CLSM images of whole kidney slices from normal and AKI mice with i.v. injection of **DHU-AKI-3** (10 mg/kg, 200 μL), scale bars = 2 mm. (j) The enlarged view in R1 and R2 regions, respectively. T: renal tubule, G: glomerulus, scale bars = 20 μm. (k) TEM images of the kidney in normal and AKI mice injected with **DHU-AKI-3** (10 mg/kg, 200 μL). White arrows indicate the precipitated CA, scale bars = 5 μm.

Encouraged by the excellent performance of **DHU-AKI-3**  *in vitro*, we further studied its feasibility for diagnosing early-stage AKI in mouse models. Ischemia-reperfusion (IR) injury is a common cause of AKI, especially in the field of renal transplantation [38]. Accurately diagnosing IR-induced kidney injury within a short time using conventional methods remains challenging, however. We constructed a unilateral kidney IR injury model to evaluate the capability of **DHU-AKI-3** for detecting early-stage AKI (Fig. 5a). We subjected the left kidney of mice to ischemia for 45 min by clamping the kidney pedicle using a mini aorta clamp, followed by reperfusion. We then intravenously injected **DHU-AKI-3** at 24 h postoperation and conducted a longitudinal ultrasound and PA imaging at different time points after the **DHU-AKI-3** injection. As shown in Fig. 5b and c, the PA signal in the kidneys of the sham operation group (b-1) was close to the background (0 h) at different time points postinjection (0.5, 1, 2, 8, 12, 24, 48, 60, and 72 h). In addition, we did not observe any PA signal enhancement in the right kidney (normal) of the IR-induced AKI mice model (b-2). We detected, however, an obvious PA signal in the left kidney (IR injury, b-2) at 1 h postinjection of **DHU-AKI-3**, which showed a long retention time, approximately 2.6-fold higher than the right kidney (normal, b-2) at 60 h. Significantly, the blood biochemical analysis showed that the SCr value of IR mice was approximately 2-fold higher than the control and sham operation groups, whereas BUN and uric acid (UA) levels did not show any significant differences (Fig. 5d–f). This result indicated that conventional clinical methods were not able to accurately diagnose unilateral kidney IR injury. By contrast, **DHU-AKI-3** enabled noninvasive, long-term monitoring of early-stage kidney injury with high specificity.

To validate the critical role of the activatable precipitation strategy in the long-term monitoring of kidney injury *in situ*, we selected the control compound **DHU-AKI-4** (lacking the CA moiety) for comparative study. Compared with the saline group, the **DHU-AKI-4** group showed weak PA signal enhancement of MB (690 nm, 1.5-fold) in the kidney only at 0.5 h post-treatment, which was rapidly cleared within 2 h (Fig. S18), indicating the rapid metabolism and short half-life of released MB *in vivo* [39]. In comparison, **DHU-AKI-3** showed obvious advantages for *in situ* long-term kidney monitoring.

### The metabolic pathways in the kidneys

Having validated that **DHU-AKI-3** was metabolized primarily in the kidneys and could diagnose early-stage AKI, we further investigated the metabolic pathways of **DHU-AKI-3** in the kidneys (Fig. 5g). Hematoxylin and eosin (H&E) staining of the renal tissue showed normal renal tubule and glomerulus morphology in the control group without cisplatin treatment (Fig. 5h). After nephrotoxic cisplatin administration, however, the injured renal tubular epithelial cells showed mild atrophy, loss of brush border, and even ballooning degeneration. The renal tubule is the kidney’s first line of defense against nephrotoxins [40,41]. Next, we dissected the kidneys at different time points after injection of **DHU-AKI-3** for fluorescence imaging and TEM analysis. The highly hydrophilic **DHU-AKI-3** was filtered out by the glomerulus and reabsorbed through renal tubules for kidney accumulation, and eventually metabolized into the urine through the normal kidney without enhanced fluorescence. Compared with the healthy control group, the fluorescence intensity of MB was enhanced only in the renal cortex of AKI mice (Fig. 5i). An enlarged view of the renal cortex (R1 and R2) clearly showed a strong fluorescence signal of MB in the renal tubular but not in the glomerulus, which illustrated that the renal clearable **DHU-AKI-3** was effectively activated in the damaged renal tubules (Fig. 5j). Furthermore, TEM images showed large precipitation in the proximal tubular epithelial capillary cavity of the mice after **DHU-AKI-3** injection (Fig. 5k). This phenomenon indicated the release of precipitated CA and further explained the *in situ* long retention time of the PA signal. These results robustly demonstrated that renal clearable **DHU-AKI-3** was capable of diagnosing early-stage AKI through the integration of *in situ* and *in vitro* detection with crosstalk-free multiplexed output.

### Integration of *in situ* and *in vitro* diagnosis by crosstalk-free multiplexed signal output

**Figure 6.**
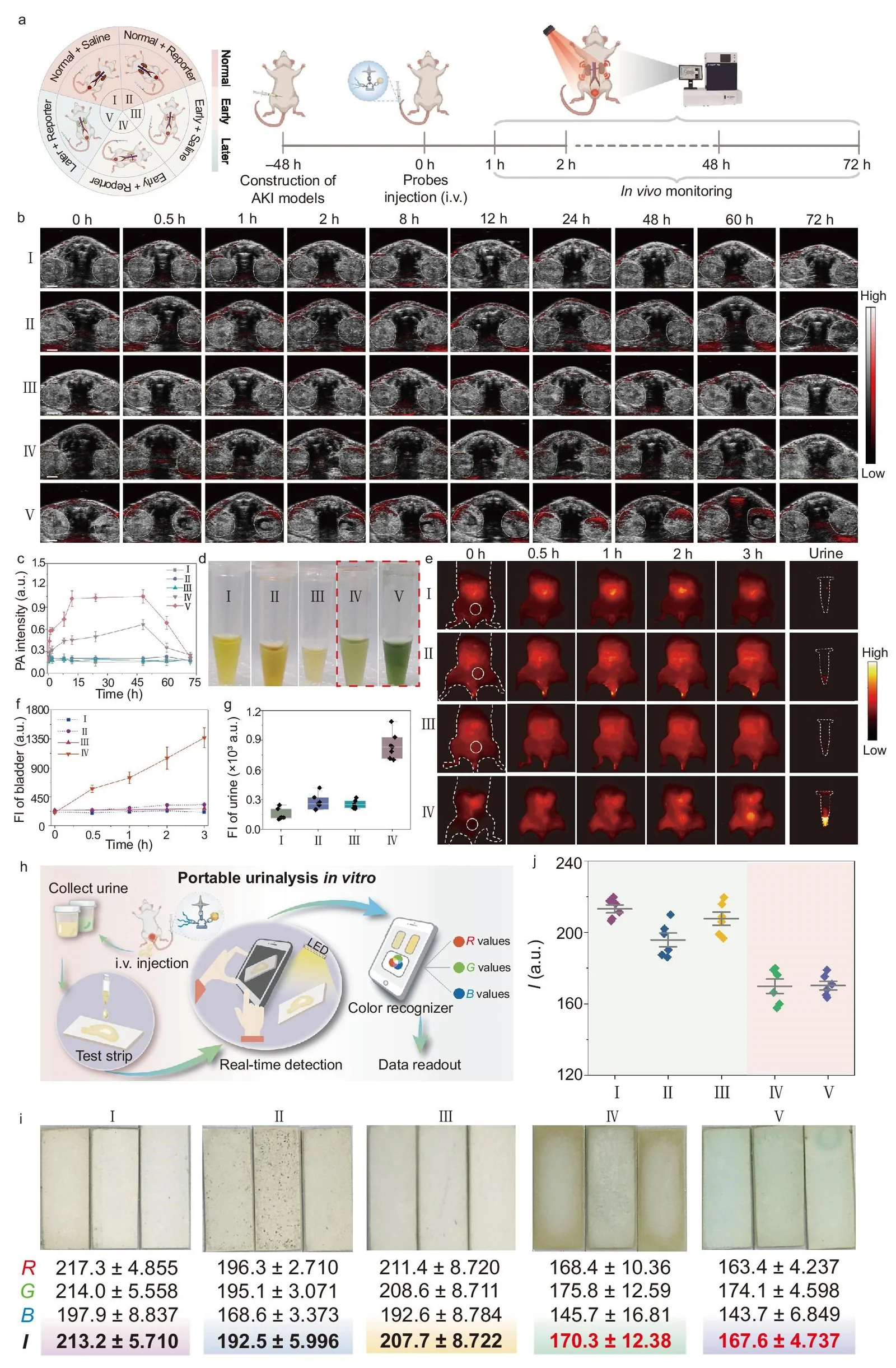
Integration of *in situ* and *in vitro* detection by crosstalk-free multiplexed signal output. (a) Schematic illustration of a two-pronged strategy. (b) Representative PA images of different mice (I: healthy mice + saline; II: healthy mice + **DHU-AKI-3**; III: early AKI mice + saline; IV: early AKI mice + **DHU-AKI-3**; V: later AKI mice + **DHU-AKI-3**) after i.v. injected with saline or **DHU-AKI-3** (10 mg/kg, 200 μL) at different post-treatment time points (0, 0.5, 1, 2, 8, 12, 24, 48, 60, and 72 h), scale bars = 2 mm. (c) The dynamic PA intensity of kidneys in (b). (d) Picture of urine collected at 3 h post-injection in (b). (e) Fluorescent images of the bladder in different groups of mice after i.v. injected with saline or **DHU-AKI-3** at different post-injection time points (0, 0.5, 1, 2 and 3 h), and collected urine at 3 h post-injection, followed by fluorescence imaging (*n =* 6). The fluorescence intensity of (f) bladder and (g) urine in (e). (h) Schematic diagram of smartphone-assisted portable urinalysis *in vitro*. (i) The photographs of the test strip with different groups of urine were captured by the camera of a smartphone, and automatically translated into RGB values by the Color Recognition software. (j) The color intensity of pictures from test strips with different groups of urine.

We studied the capability of **DHU-AKI-3** for two-pronged diagnosis of early-stage AKI using a cisplatin-induced AKI mouse model. Different stages of AKI mice were intravenously injected with saline (control group) and **DHU-AKI-3**, respectively (Fig. 6a). We performed ultrasound/PA imaging at different time points after administration of **DHU-AKI-3**. For the healthy groups (I and II) without the cisplatin treatment, the PA signal in the kidneys remained consistent with the background throughout the tracking process (Fig. 6b). The same was true for the PA signal in the AKI mice after injection of saline (group III). For the early-stage AKI group (group IV), however, the PA intensity of CA rapidly increased in kidneys at 0.5 h after injection, which was ∼1.6-fold higher than the control time (0 h), further showing the long retention time of the CA signal in kidneys (∼60 h). Moreover, the PA signal in the kidneys of late-stage AKI mice (group V) followed a similar trend, but with stronger PA intensity (∼2.5-fold enhancement at 0.5 h postinjection) (Fig. 6c). Because more ROS was produced in the late-stage damaged kidneys, more abundant CA was released and precipitated *in situ*. These results indicated that **DHU-AKI-3** achieved long-term monitoring of early-stage AKI *in situ*.

To further confirm the integration diagnosis of **DHU-AKI-3** for *in vivo* and *in vitro* detection with crosstalk-free duplex signal output, we collected the urine samples from each group (I–V) of mice at 3 h post-treatment of **DHU-AKI-3**. As shown in Fig. 6d, the urine color of the control groups (I–III) all showed yellow, whereas the urine color of groups IV and V changed from yellow to visible light green and dark green, respectively. Further HR-MS detection of urine verified the presence of MB (see Fig. S19 in SI). These results indicated that blue MB was excreted into the urine of groups IV and V, which were consistent with the PA imaging data *in situ*. This phenomenon of urine color changes verified the feasibility of portable urinalysis *in vitro* by colorimetric analysis. We then measured the MB signal in the bladder and urine of different groups by *in situ* fluorescence imaging and *in vitro* colorimetric methods (Fig. 6e). We found a negligible fluorescence signal of MB in the bladder of control groups I and II at different time points after injection with saline and **DHU-AKI-3**. Similarly, the fluorescence signal in urine at 3 h post-treatment showed no significant difference (Fig. 6f and g). Compared with the early-stage AKI mice (group III) after injection of saline, we detected a statistically increased fluorescence in the bladder of group IV in a time-dependent manner and showed a ∼3.5-fold enhancement in urine at 3 h postinjection of **DHU-AKI-3**. This high consistency of urinalysis *in vitro* and PA imaging *in situ* ensured the accuracy of urine diagnosis.

To achieve a more convenient detection application, we developed a smartphone-assisted colorimetric urinalysis *in vitro* for portable diagnosis of early-stage AKI (Fig. 6h). We collected urine samples from all mice of different groups at predetermined times and dripped the samples on the surface of white test strips. The strips showed obvious color changes that were visible to the naked eye, thus facilitating visual real-time monitoring *in vitro*. We further combined the camera of the smartphone and the Color Recognition software to realize convenient detection. The pictures of the test strip were recognized by software that automatically translated the results into *R* (Red), *G* (Green), and *B* (Blue) values. We calculated the color intensity of pictures according to the formula *I* = 0.3*R* + 0.59*G* + 0.11*B* [42]. As shown in Fig. 6i and j, groups I–III showed higher color intensity (*I*), whereas the *I* value of urine from groups IV and V was statistically significantly decreased, which indicated the excretion of blue MB from the kidney into urine. Accordingly, the mutual authentication of duplex optical signal output (PA signal of CA *in situ* and fluorescence signal of MB in bladder) validated the accuracy of portable urinalysis *in vitro*. Thus, the unimolecular dual-reporter probe **DHU-AKI-3** could accurately diagnose early-stage AKI by integration of *in situ* long-term monitoring and portable urinalysis *in vitro* with crosstalk-free multiplexed output. Overall, this method is a promising tool for visual analysis of the AKI state directly in the urine, matching the demands of future point-of-care testing.

## CONCLUSION

In summary, we reported a unimolecular dual-reporter probe, **DHU-AKI-3**, with crosstalk-free multiplexed output for precise and portable diagnosis of early AKI. **DHU-AKI-3** showed a renal enrichment effect and could be specifically activated by ROS, thus ensuring high sensitivity for early AKI diagnosis. Its rapid response facilitates PA signal accumulation to a detectable threshold within a short time, allowing for effective long-term *in situ* monitoring. The ‘turn-on’ NIRF signal (MB) of **DHU-AKI-3** after being stimulated by ROS reduced the interference of other reactive species. The integration of *in situ* and *in vitro* detection benefited from the distinct metabolic characteristics of crosstalk-free multiplexed signals. In particular, by directly linking the urine signal to the same activation event occurring in the injured kidney, this unimolecular dual-reporter probe established a physiological bridge between urine readout and renal pathology and provided an accurate and portable tool for the diagnosis of early-stage AKI, offering a practical route for point-of-care screening, bedside monitoring, and longitudinal follow-up in high-risk patients (e.g. ICU, transplantation, nephrotoxic chemotherapy). As a result, this work offers a versatile two-pronged strategy for rationally designing probes that not only allow for the long-term monitoring of diseases *in situ* and portable diagnosis *in vitro* but also make simultaneous visual readout of the AKI state possible, thus holding potential for further point-of-care testing.

## METHODS

### Materials, reagents and characterization methods

The source of materials and reagents, and characterization methods used can be found in Supplementary Information.

### Cell culture and confocal laser scanning microscopy (CLSM) imaging

Human proximal tubular epithelial cell line (HK-2) was cultured in basic Minimum Essential Medium (MEM) supplemented with 10% fetal bovine serum (FBS) and 1% Penicillin-Streptomycin. The cells were incubated at 37°C under 5% CO_2_ and split with trypsin/EDTA solution (0.25%) as recommended by the manufacturer.

Cells (5 × 10^8^ per mL) were separately plated on 14 mm glass coverslips and allowed to adhere for 12 h. The stock solution of reporters in DMF (5 mM) was diluted with phosphate buffered saline (PBS, 10 mM, pH 7.4) with final concentration of 10 μM. The cells were then incubated with different analytes for a pre-set time at 37°C. After incubation, the cells were washed two times with PBS. CLSM imaging was performed on ZEISS LSM 880 Confocal Laser Scanning Microscope with a 60 × oil-immersion objective lens for cells. Red channel: 700 ± 50 nm, *λ*_ex_ = 633 nm.

### *In vivo* fluorescence imaging

Healthy mice and cisplatin-induced AKI mice model were all given an i.v. injection of **DHU-AKI-3** (10 mg/kg body weight, 200 μL) and saline (control). Then, all of the mice were anaesthetized and the imaging experiments were implemented on a self-constructed small animal *in vivo* imaging system with 635 nm excitation laser and Andor EMCCD 597. The bladder (ventral side) of mice images were recorded at 0, 0.5, 1, 2 and 3 h.

### Urinalysis *in vitro*

The individual differences of mice urine may be large, and therefore normalization between urine samples is required. In this study, we normalized the urine volume and injected dose as far as possible. The urine samples were collected from different experimental groups of living mice at 3 h post-treatment with various compounds. On the one hand, the collected urine samples were detected by fluorescence imaging with 635 nm excitation laser. On the other hand, urine samples were dropped onto the test strips followed by taking pictures of the test strip using the camera of smartphone. Then, the Color Recognition software immediately readout *R* (Red), *G* (Green), *B* (Blue) values of urine pictures. Each group has six parallel replicates.

### Ethical statements

All animal experiments were conducted in accordance with the Chinese Regulations for the Administration of Affairs Concerning Experimental Animals (SYXK-2020–0018) and were approved by the Institutional Animal Care and Use Committee of Donghua University (DHUEC-NSFC-2024–44). All procedures complied with the Guide for the Care and Use of Laboratory Animals and conformed to internationally accepted guidelines (National Institutes of Health Guide for the Care and Use of Laboratory Animals, Publication No. 85–23, revised 1996). Efforts were made to minimize animal suffering and to reduce the number of animals used.

## Supplementary Material

nwag367_Supplemental_Files
