# Supplementary material for: Unimolecular dual-reporter probes for early kidney injury diagnosis through synchronous in situ imaging and portable urinalysis
Source: Natl Sci Rev. 2026 Jun 12;13(16):nwag367. doi: 10.1093/nsr/nwag367 (PMC13499234; doi:10.1093/nsr/nwag367)
Supplement: Supplementary file 1 [file nwag367_supplemental_files.zip › 20260611-SI-NSR_MS-2025-2343.R1-LLY.pdf]

Supporting Information

Unimolecular dual-reporter probes for early kidney injury diagnosis through synchronous *in situ* imaging and portable urinalysis

Lingyan Liu[1,#], Feiyang Liu[2,#], Qihang Ding[3,*], Shasha Wang[1], Peng Wei[1,*], Jong Seung Kim[3,*], and Tao Yi[1,*]

[1] State Key Laboratory of Advanced Fiber Materials, College of Chemistry and Chemical Engineering, Donghua University, Shanghai 201620, China.

[2] Guangxi Key Laboratory of Special Biomedicine, School of Medicine, Guangxi University, Nanning, 530004 China.

[3] Department of Chemistry, Korea University, Seoul, 02841, Korea.

*Email: dingqihang@whu.edu.cn; weipeng@dhu.edu.cn; jongskim@korea.ac.kr; yitao@dhu.edu.cn.

[#]Equally contributed to this work.

Content

1 Materials and Experiment Section 2

1.1 Chemicals and bioreagents 2

1.2 Materials characterization 2

1.3 General procedure for the synthesis of compounds 3

1.4 reparation of reporters and analytes 9

1.5 Spectroscopic studies *in vitro* 10

1.6 Cytotoxicity experiments 10

1.7 Renal cell apoptosis model 10

1.8 Establishment of different AKI mice models 11

1.9 Bio-distribution studies of DHU-AKI-3 in mice 11

1.10 Ultrasonic/photoacoustic imaging *in vivo* 11

1.11 Blood routine analysis 12

1.12 Histopathological studies 12

1.13 Statistical analysis 12

1.14 Data availability 12

2 Additional tables 14

3 Additional images and figures 16

4 NMR and HR-MS spectra 25

5 References 35

1 Materials and Experiment Section

1.1 Chemicals and bioreagents

All starting materials were obtained from commercial suppliers and used without further purification. Notably, methylene blue, and sodium hydrosulfite were purchased from Adamas-beta and Sun Chemical Technology (Shanghai) Co., LTD., respectively. Cell counting kit-8 (CCK-8) and Annexin V-FITC apoptosis detection kit were purchased from Beyotime and Sangon Biotech (Shanghai) Co., Ltd., respectively. HK-2 cell line was purchased from Procell Life Science & Technology Co., Ltd.

All experiments were performed in compliance with the relevant laws and institutional guidelines. All animal experiments were performed according to the guidelines of the Institutional Animal Care and Use Committee.

1.2 Materials characterization

^{1}H NMR (400 MHz) and ^{13}C NMR (100 MHz) were obtained on a Bruker AV400 nuclear magnetic resonance spectrometer. Proton chemical shifts are reported in parts per million downfield from tetramethylsilane (TMS), with tetramethylsilane (δ = 0.0 ppm) or DMSO-d_6 (2.50 ppm for ^{1}H and 39.52 ppm for ^{13}C) as the chemical shift standard. High-resolution mass spectra (HRMS) were carried out on a Bruker Micro TOF II instrument with the electro-spray ionization (ESI) technique and direct injection method. UV-visible spectra were performed using a Shimadzu UV-2550 spectrometer. Steady-state fluorescence spectra and time-dependent studies were obtained on an Edinburgh instrument FLS-1000 spectrometer with a Xe lamp as an excitation source. High contrast transmission electron microscopy (HCTEM) was performed using a Hitachi HT7800 transmission electron microscope with an accelerating voltage of 100 kV. Dynamic light scattering (DLS) and zeta potential analysis were performed on a Nano-ZS Zetasizer (Malvern, UK). Confocal laser scanning microscopy (CLSM) was performed on ZEISS LSM 880 Confocal Laser Scanning Microscope with a 60 × oil-immersion objective lens. The cellular fluorescence was measured using a Coulter

Gallios flow cytometer (Beckman). *In vivo* imaging was collected using Bruker multi-angle optical *in vivo* imaging system (*In Vivo* Xtreme) with a Xe lamp as an excitation source. Ultrasonic/photoacoustic imaging was performed with a LAZR commercial instrument (FujiFilm VisualSonics) equipped with a 21 MHz-centered transducer. Another 3D optoacoustic Imaging was performed under a 3D optoacoustic imaging system (LOIS-3D, TomoWave Suzhou Medical Imaging Co.,Ltd, China).

1.3 General procedure for the synthesis of compounds

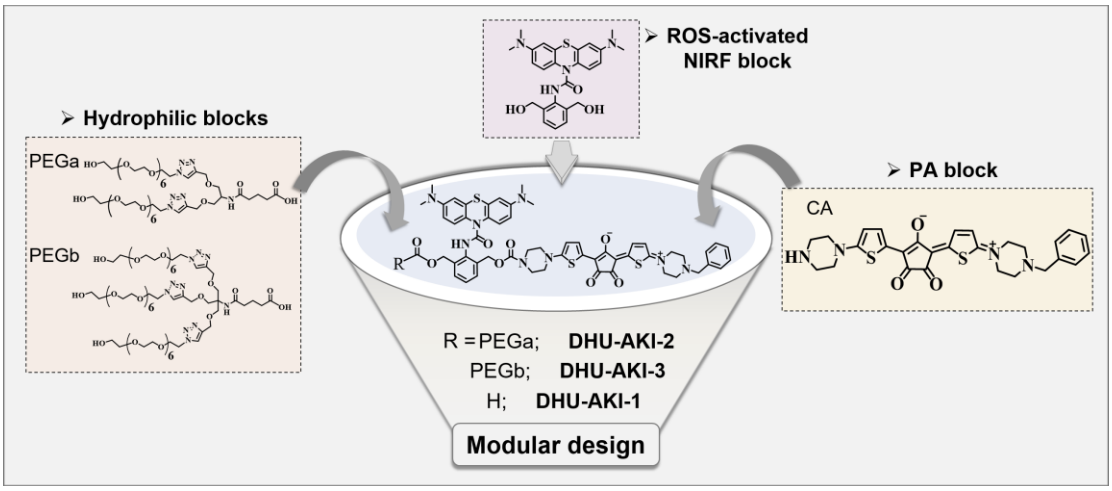

Scheme S1. The modular design and chemical structure of probes.

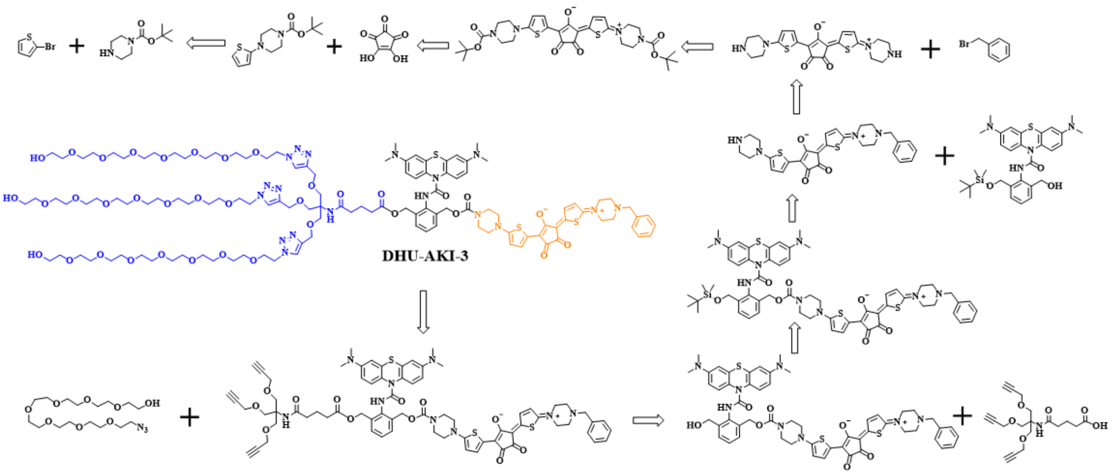

Scheme S2. The retrosynthetic analysis of **DHU-AKI-3**.

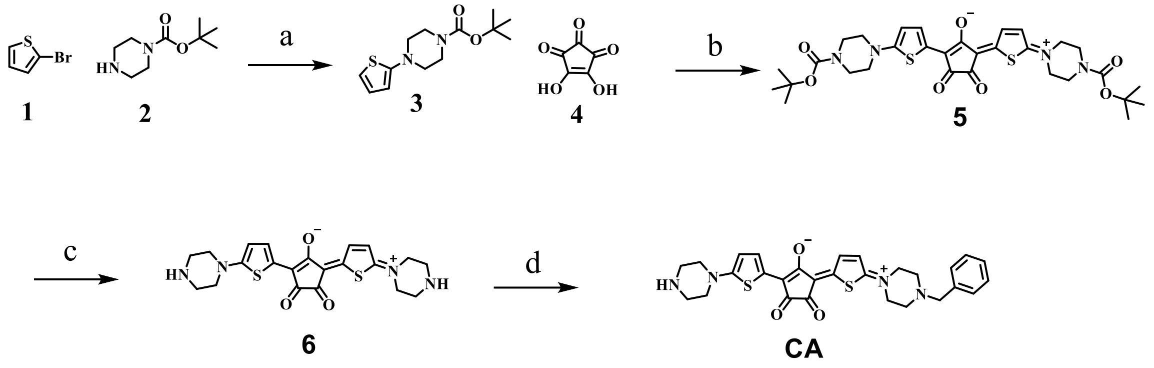

Scheme S3. The synthetic route of asymmetric croconic acid dye (CA). Reagents and conditions: (a) CuI, copper powder, K_3PO_4, N, N-Dimethyl ethanolamine (DMAE), 80°C, 48 h, 30%; (b) toluene/1-butanol, reflux., 1 h, 84%; (c) trifluoroacetic acid (TFA), dichloromethane (DCM), r.t., 3 h; (d) benzyl bromide, K_2CO_3, dimethyl formamide (DMF), r.t., 2 h, 25%.

Synthesis of compound 3. Compound **1** (2.4 mL, 24.6 mmol), CuI (700 mg, 3.6 mmol), copper powder (235 mg, 3.6 mmol), K_3PO_4 (10.44 g, 49.2 mmol) and Compound **2** (6.87 g, 36.9 mmol) were dissolved into N, N-Dimethyl ethanolamine (DMAE, 30 mL) and then heated at 80 °C for 48 h under the protection of N_2. After that, the reaction mixture was cooled to room temperature and added with water (100 mL). The solid was removed by filtration and the solution was extracted by ethyl acetate (3 × 50 mL). The combined organic solution was washed by brine (1 × 100 mL), dried over Na_2SO_4 and evaporated in vacuum to give the crude product which was further purified by silica gel column chromatography to give compound **3** as a white solid (1.28 g, 5.96 mmol, 30%). ^{1}H NMR (400 MHz, $CDCl_3$) δ 6.81 (dd, J = 5.2, 4.0 Hz, 1H), 6.67 (d, J = 5.6 Hz, 1H), 6.19 (d, J = 3.6 Hz, 1H), 3.59 (t, J = 5.2 Hz, 4H), 3.11 (t, J = 5.0 Hz, 4H), 1.50 (s, 9H). ^{13}C NMR (100 MHz, $CDCl_3$) δ 159.2, 154.7, 126.2, 113.3, 106.6, 80.1, 52.0, 43.4, 28.5.

Synthesis of compound 5. Compounds **4** (265 mg, 1.86 mmol) and **3** (1 g, 3.73 mmol) were dissolved in the mixture of toluene (15) and 1-butanol (15 mL) and heated to reflux for 1 h under the protection of N_2. After that, the reaction mixture was cooled to room temperature and evaporated in vacuum. The crude residue was purified by silica gel column chromatography to give the product as a brown solid (1 g, 1.56 mmol, 84%). ^{1}H NMR (400 MHz, $CDCl_3$) δ 8.80 – 8.63 (m, 2H), 6.56 (s, 2H), 3.65 (s, 16H), 1.49 (s, 18H).

Synthesis of croconic acid (CA). To the solution of compound **5** (1 g, 1.56 mmol) in 16 mL of dichloromethane (DCM) was added 4 mL of trifluoroacetic acid (TFA). The mixture was stirred at room temperature for 3 h. The reaction solvent was removed in vacuum to give the crude product which was used directly in next step without further purification. The crude residue dissolved in 10 mL of dimethyl formamide (DMF) and K_2CO_3 (645 mg, 4.67 mmol) was stirred at room temperature for 10 min, and benzyl bromide (266 mg, 1.56 mmol) dissolved in 2 mL of DMF was dropwise added. After that, the mixture was stirred at room temperature for 2 h, thin layer chromatography (TLC) suggested the completion of the reaction. The reaction solution was removed in vacuum and purified by silica gel column chromatography to give the croconic acid (**CA**) dye as a black solid (210 mg, 25%). ^{1}H NMR (400 MHz, DMSO-d_6) δ 9.1 (s, 1H), 8.46 (s, 1H), 7.38 - 7.24 (m, 6H), 6.89 (s, 1H), 3.84 (s, 4H), 3.59 (s, 2H), 3.10 (q, J = 7.2 Hz, 8H), 2.62 (s, 4H). HR MS (ESI): m/z $[M + H]^+$ calcd for $C_{28}H_{29}N_4O_3S_2^+$: 533.1676, found 533.1683.

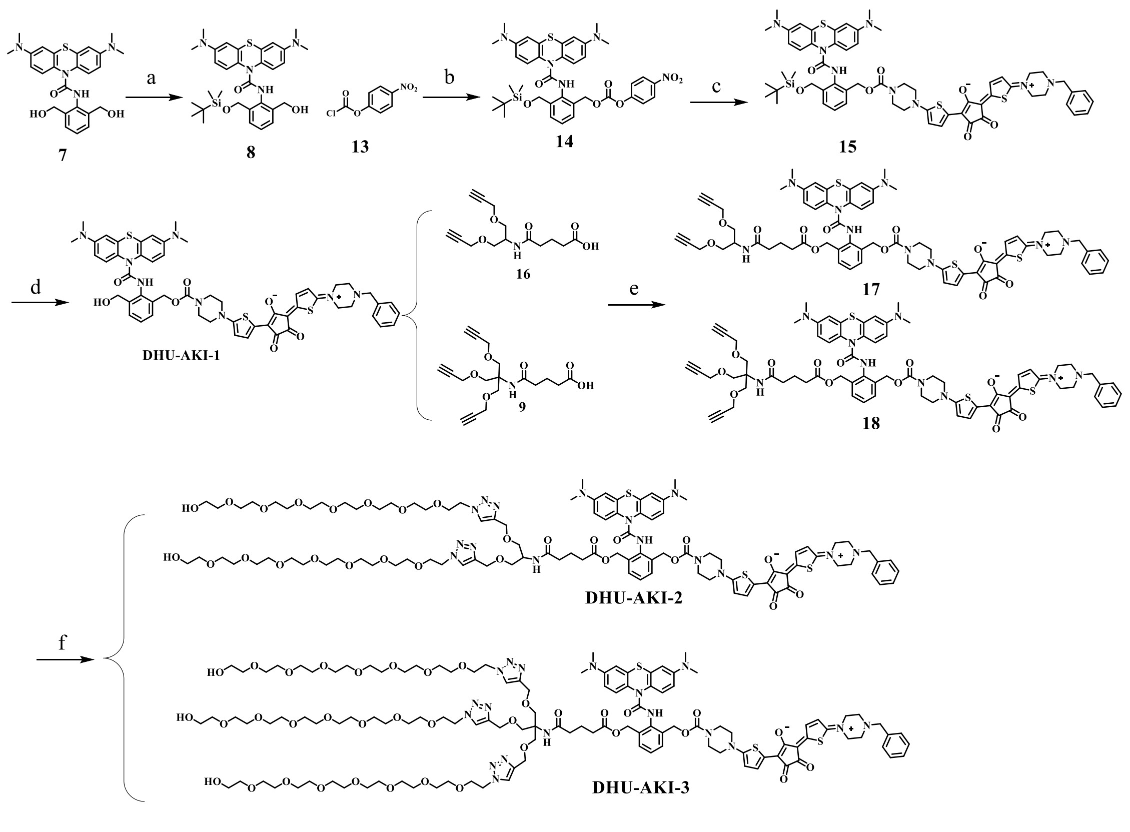

Scheme S4. The synthetic route of the renal reporters (DHU-AKI-2 and DHU-AKI-3). Reagents and conditions: (a) imidazole, *t*-butyldimethylsilyl chloride (TBSCl), DMF, 0 °C to r.t., 5 h, then TsOH, MeOH, r.t., 30 min, 55%; (b) triethylamine (TEA),

DCM, 0 °C to r.t., 2 h; (c) **CA,** TEA, DMF, r.t., 3 h, 36%; (d) TsOH, MeOH, r.t., 1 h, 67%; (e) EDCI, DMAP, DCM, r.t., 2 h, 76-80%; (f) sodium ascorbate, $CuSO_4$•$5H_2O$, THF/H_2O, r.t., 2 h, 31%.

Synthesis of compound 15. To a 50 mL round bottom flask was added compound **8** (500 mg, 0.86 mmol), trimethylamine (Et_3N, 0.36 mL, 2.59 mmol) and DCM (5 mL). Compound **13** (209 mg, 1.04 mmol) dissolved in DCM (3 mL) was added dropwise at 0 °C, and then the reaction mixture was stirred at room temperature for 2 h. TLC showed the completion of the reaction. The reaction mixture was washed with 1 N HCl solution (10 mL), dried over sodium sulfate, filtered and concentrated to give the intermediate which was used directly in next step. To the intermediate with DMF (5 mL) and Et_3N (0.36 mL, 2.59 mmol) was added **CA** (458 mg, 0.86 mmol), the mixture was then stirred at room temperature for 3 h. The reaction mixture was poured into H_2O (100 mL), and extracted with ethyl acetate (EA, 20 mL × 3). The combined organic layers were dried with Na_2SO_4 and concentrated in vacuum to give the crude product which was further purified by silica gel column chromatography to give compound **15** as a black solid (350 mg, 35.8%). ^{1}H NMR (400 MHz, $CDCl_3$) δ 8.67 (s, 2H), 8.04 (d, J = 8.8 Hz, 2H), 7.43 (t, J = 8.4 Hz, 3H), 7.33 (d, J = 8.0 Hz, 4H), 7.04 (s, 1H), 6.86 (d, J = 8.8 Hz, 2H), 6.70 (s, 2H), 6.59 (d, J = 8.4 Hz, 2H), 6.43 (s, 1H), 5.12 (s, 2H), 4.77 (s, 2H), 3.71 – 3.30 (m, 14H), 2.91 (s, 12H), 2.66 (s, 4H), 0.89 (s, 9H), 0.07 (s, 6H).

Synthesis of DHU-AKI-1. Compound **15** (350 mg, 0.31 mmol) was dissolved in 10 mL of MeOH and *p*-toluenesulfonic acid (53 mg, 0.31 mmol) was added. The mixture was stirred at room temperature for 1 h, TLC suggested the completion of the reaction. The reaction solution was removed in vacuum and purified by silica gel column chromatography to give **DHU-AKI-1** as a light blue solid (210 mg, 66.7%). ^{1}H NMR (400 MHz, $CDCl_3$) δ 8.86 – 8.62 (m, 2H), 7.52 - 7.47 (m, 3H), 7.38 - 7.28 (m, 7H), 7.16 (s, 1H), 6.70 – 6.57 (m, 5H), 6.38 (s, 1H), 5.00 (s, 2H), 4.73 (s, 2H), 3.72 (s, 5H), 3.60 (s, 2H), 3.52 (s, 3H),3.23 (s, 4H), 2.92 (s, 12H), 2.66 (s, 4H). HR MS (ESI): m/z $[M + H]^+$ calcd for $C_{54}H_{55}N_8O_7S_3^+$: 1023.3350, found 1023.3347.

Synthesis of compound 17. To the mixture of compounds **DHU-AKI-1** (50 mg, 0.049 mmol) and **16** (15 mg, 0.054 mmol) in 3 mL of DCM was added EDCl (14 mg,

0.073 mmol) and DMAP (6 mg, 0.049 mmol), the mixture was stirred at room temperature for 2 h. TLC suggested the completion of the reaction, H_2O (20 mL) was added to the reaction solution, extracting with DCM (10 mL × 3). The combined organic layers were dried with Na_2SO_4 and concentrated in vacuum to give the crude product which was further purified by silica gel column chromatography to give compound **17** as a black solid (50 mg, 79.5%). ^{1}H NMR (400 MHz, $CDCl_3$) δ 8.67 (s, 2H), 7.48 - 7.27 (m, 9H), 6.99 (s, 1H), 6.70 – 6.56 (m, 5H), 6.40 (s, 1H), 5.98 (d, *J* = 7.2 Hz, 1H), 5.25 (s, 2H), 5.07 (s, 2H), 4.25 (s, 1H), 4.12 (s, 4H), 3.68 - 3.26 (m, 18H), 2.91 (s, 12H), 2.63 (s, 4H), 2.41 (d, *J* = 12.4 Hz, 4H), 2.20 (d, *J* = 6.4 Hz, 2H), 1.96 (d, *J* = 6.4 Hz, 2H).

Synthesis of DHU-AKI-2. To the solution of compounds **17** (50 mg, 0.039 mmol) and **12** (38 mg, 0.097 mmol) in a mixture of THF (4 mL) and H_2O (1 mL) was added sodium ascorbate (3 mg, 0.016 mmol) and $CuSO_4 \bullet 5H_2O$ (1.9 mg, 0.008 mmol). The mixture was stirred at room temperature under N_2 atmosphere for 2 h. TLC suggested the completion of the reaction, the solvent was then removed in vacuum and purified by dialysis for 48 h. The product was lyophilized to give **DHU-AKI-2** as a black solid (25 mg, 31%). ^{1}H NMR (400 MHz, $CDCl_3$) δ 8.73 (s, 2H), 7.76 (s, 2H), 7.31 (d, *J* = 22.1 Hz, 11H), 6.69 (d, *J* = 28.7 Hz, 6H), 5.12 (s, 4H), 4.57 (d, *J* = 34.5 Hz, 4H), 3.75 (d, *J* = 87.6 Hz, 83H), 2.93 (s, 12H), 2.67 (s, 4H), 2.40 (s, 2H), 2.23 (s, 2H), 1.94 (s, 2H). HR MS (ESI): m/z $[M + H]^+$ calcd for $C_{100}H_{138}N_{15}O_{27}S_3^+$: 2076.9077, found 2076.9060.

Synthesis of compound 18. The synthetic method of compound **18** is similar to that of compound **17**. **18** was produced as a black solid (50 mg, 75.5%). ^{1}H NMR (400 MHz, $CDCl_3$) δ 8.85 – 8.63 (m, 2H), 7.49 - 7.42 (m, 3H), 7.38 - 7.28 (m, 6H), 6.91 (s, 1H), 6.70 - 6.57 (m, 5H), 6.39 (s, 1H), 5.74 (s, 1H), 5.27 (s, 2H), 5.05 (s, 2H), 4.13 (d, *J* = 1.6 Hz, 6H), 3.83 (s, 6H), 3.76 - 3.60 (m, 10H), 3.36 (s, 2H), 3.21 (s, 2H), 2.92 (s, 12H), 2.66 (s, 2H), 2.43 (s, 5H), 2.21 (t, *J* = 7.0 Hz, 2H), 1.98 - 1.90 (m, 2H), 1.71 (s, 2H).

Synthesis of DHU-AKI-3. The synthetic method of **DHU-AKI-3** is similar to that of **DHU-AKI-2**. **DHU-AKI-3** was produced as a black solid (40 mg, 42.6%). ^{1}H NMR

(400 MHz, DMSO-d_6) δ 8.53 (s, 2H), 8.01 (s, 3H), 7.36 (s, 13H), 6.75 – 6.57 (m, 4H), 5.14 (s, 2H), 5.02 (s, 2H), 4.49 (s, 12H), 3.80 (s, 8H), 3.50 (s, 102H), 2.87 (s, 12H), 2.60 (s, 4H), 2.35 (s, 2H), 2.14 (s, 2H), 1.74 (s, 2H). HR MS (ESI): m/z $[M + H]^+$ calcd for $C_{120}H_{175}N_{18}O_{36}S_3^+$: 2540.1603, found 2540.1600.

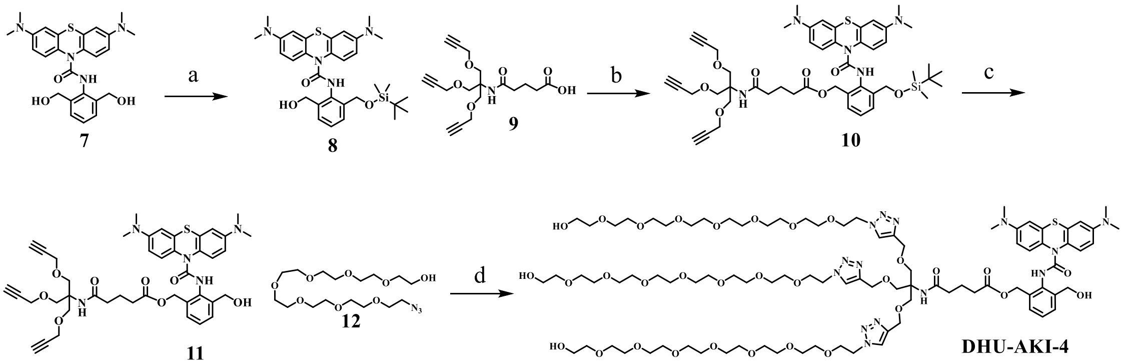

Scheme S5. The synthetic route of control compound (DHU-AKI-4). Reagents and conditions: (a) imidazole, t-butyldimethylsilyl chloride (TBSCl), DMF, 0 °C to r.t., 5 h, then p-toluenesulfonic acid (TsOH), methanol (MeOH), r.t., 30 min, 55%; (b) 1-ethyl-3-(3-dimethylaminopropyl) carbodiimide (EDCI), dimethylaminopyridine (DMAP), DCM, r.t., 2 h; (c) TsOH, MeOH, r.t., 1 h, 83%; (d) sodium ascorbate, $CuSO_4{\bullet}5H_2O$, tetrahydrofuran (THF)/H_2O, r.t., 2 h, 44%.

Synthesis of compound 11: To the mixture of compound **8** (330 mg, 0.57 mmol, prepared according to our previous method [1]) and compound **9** (200 mg, 0.57 mmol) in 10 mL of DCM was added 1-ethyl-3-(3-dimethylaminopropyl) carbodiimide (EDCl, 164 mg, 0.86 mmol) and dimethylaminopyridine (DMAP, 70 mg, 0.57 mmol), the mixture was stirred at room temperature for 2 h. TLC suggested the completion of the reaction, H_2O (50 mL) was added to the reaction solution, extracting with DCM (20 mL × 3). The combined organic layers were dried with Na_2SO_4 and concentrated in vacuum to give the crude product compound **10** which was used directly in next step. TsOH (99 mg, 0.57 mmol) was added to the crude residue dissolved in 10 mL of MeOH. The mixture was then stirred at room temperature for 1 h, TLC suggested the completion of the reaction. The reaction solvent was removed in vacuum and the crude product was purified by silica gel column chromatography to give compound **11** as a light blue solid (380 mg, 83.4%). ^1H NMR (400 MHz, $CDCl_3$) δ 7.47 - 7.42 (m, 3H), 7.30 - 7.26 (m, 2H), 7.21 (t, J = 7.6 Hz, 1H), 6.75 (d, J = 2.8 Hz, 2H), 6.65 (dd, J = 8.8, 2.8 Hz, 2H), 5.69 (s, 1H), 5.00 (s, 2H), 4.62 (s, 2H), 4.12 (d, J = 2.4 Hz, 6H), 3.81 (s,

6H), 2.94 (s, 12H), 2.43 (t, J = 2.2 Hz, 3H), 2.12 (dt, J = 29.2, 7.2 Hz, 4H), 1.81 - 1.74 (m, 2H). ^{13}C NMR (100 MHz, $CDCl_3$) δ 172.8, 172.2, 156.4, 149.3, 138.3, 135.5, 134.8, 131.8, 131.6, 130.1, 128.2, 127.6, 127.0, 111.4, 111.1, 79.6, 74.8, 68.6, 64.2, 62.6, 59.3, 58.8, 40.8, 35.9, 32.9, 20.7.

Synthesis of DHU-AKI-4: To the solution of compounds **11** (100 mg, 0.13 mmol) and **12** (174 mg, 0.44 mmol) in a mixture of tetrahydrofuran (THF, 4 mL) and H_2O (1 mL) was added sodium ascorbate (12.4 mg, 0.06 mmol) and $CuSO_4 \bullet 5H_2O$ (12.6 mg, 0.05 mmol). The mixture was stirred at room temperature under N_2 atmosphere for 2 h. TLC suggested the completion of the reaction, the solvent was then removed in vacuum and purified by silica gel column chromatography to give the control compound **DHU-AKI-4** as a light blue solid (110 mg, 44.2%). ^{1}H NMR (400 MHz, $CDCl_3$) δ 7.72 (d, J = 14.8 Hz, 3H), 7.46 - 7.36 (m, 4H), 7.28 (s, 1H), 7.20 - 7.13 (m, 1H), 6.74 - 6.64 (m, 4H), 6.27 (s, 1H), 5.01 (s, 2H), 4.62 (s, 2H), 4.58 - 4.50 (m, 12H), 3.87 - 3.84 (m, 6H), 3.78 (s, 6H), 3.63 - 3.59 (m, 84H), 2.94 (s, 12H), 2.12 (dt, J = 34, 7.2 Hz, 4H), 1.79 - 1.72 (m, 2H). ^{13}C NMR (100 MHz, $CDCl_3$) δ 172.8, 172.4, 156.1, 149.2, 144.7, 138.2, 135.4, 134.8, 132.1, 131.0, 129.8, 127.6, 126.8, 123.9, 111.5, 111.1, 72.7, 70.6, 70.4, 69.5, 69.0, 64.8, 64.0, 62.6, 61.8, 59.9, 50.2, 40.8, 35.8, 33.0, 20.9. HR MS (ESI): m/z $[M + H]^+$ calcd for $C_{91}H_{149}N_{14}O_{32}S^+$: 1982.0178, found 1982.0183.

1.4 Preparation of reporters and analytes

Stock solutions of reporters (5 mM) were prepared in DMF, and then diluting with phosphate buffer (PB, pH 7.4) to the test concentration. The test solutions were shaken well and placed at room temperature before recording the spectra. Other analytes were prepared in ddH_2O according to our reported procedure and details are as follows. HOCl was obtained from NaOCl solution (0.1 M). H_2O_2 was diluted from a 30 % solution. $^{\bullet}$OH (Hydroxyl radical) was generated by Fenton reaction (H_2O_2: $FeCl_2$ = 1: 10) and the concentration of $^{\bullet}$OH was equal to the concentration of H_2O_2. TBHP (tert-butyl hydroperoxide) was obtained from 70% TBHP solution in ddH_2O. ROO$^{\bullet}$ was prepared by dissolving 2,2'-azobis(2-amidinopropane) dihydrochloride in ddH_2O. NO

was prepared by dissolving SNP (sodium nitroferricyanide (III) dihydrate) in ddH_2O. O_2^- was prepared by dissolving KO_2 (potassium superoxide) in DMSO. $ONOO^-$ was prepared using 3-morpholinosydnonimine hydrochloride. *t*-BuOO$^•$ was prepared by adding TBHP in the presence of 10 equiv. of ferrous chloride and the concentration of *t*-BuOO$^•$ was equal to the TBHP concentration.

1.5 Spectroscopic studies *in vitro*

According to the published methods [2], different reporter were treated with various concentrations of ROS in PB (pH 7.4) for 30 min. Fluorescence or absorption spectra were measured on a fluorescence or ultraviolet spectrophotometer. Unless otherwise noted, the excitation wavelength was 620 nm for all fluorescent tests, and the emission wavelength was collected from 630 to 830 nm.

1.6 Cytotoxicity experiments

A cell counting kit-8 (CCK-8) was employed for this experiment to quantitatively evaluate HK-2 viability. Cells were seeded in 96-well microplates at a density of 1×10^4 cells per well, then cultured in 5% CO_2 at 37 °C for 24 h. After the cells were incubated with **DHU-AKI-3** at different concentrations (0, 5, 10, 20, 50, 80, 100, 150, 200 and 300 μM in DMF/MEM medium without FBS = 1: 100) for 24 h, 100 μL MEM containing 10% CCK-8 was added to each well of the 96-well assay plate for 4 h at 37 °C, six parallel replicates were prepared. The absorbance at 450 nm was determined using a microplate reader (Bio Tek Instruments, Inc). All samples were analyzed in triplicate.

1.7 Renal cell apoptosis model

The Annexin V-FITC apoptosis detection kit was employed for evaluation of HK-2 apoptosis. Cells were seeded in 6-well plates at a density of 1×10^6 cells per well in 2 mL medium containing 10% FBS, then cultured in 5% CO_2 at 37 °C for 24 h. After the cells were incubated with cisplatin at different concentrations (0, 300, 600 and 1000

μM) for 12 h, the cells were washed in PBS for twice and collected by trypsin treatment. Then the cells were re-suspended with 800 μL PBS, followed by the detection of flow cytometry using Annexin V-FITC apoptosis detection kit. All the experiments detected at least 20,000 cells, and these data were analyzed in the FlowJo V10.

1.8 Establishment of different AKI mice models

The drug-induced different degrees of kidney injury mice were constructed according to the previous method [3], the early and later kidney injury mice model were induced by intraperitoneal injections with different doses of cisplatin (10, and 20 mg/kg) for 48 h, respectively. To construct unilateral kidney ischemia reperfusion (IR) injury mice model, the F1 female mice (6-8 weeks old) were anesthetized with 5% chloral hydrate. The left renal pedicle was clamped completely using mini aorta clamp for 45 min and then releasing, different compounds were intravenous (i.v.) injected at 24 h post-operation. The hair of mice was removed before the subsequent imaging experiments.

1.9 Bio-distribution studies of DHU-AKI-3 in mice

Different mice models were all given an i.v. injection of **DHU-AKI-3** (10 mg/kg body weight, 200 μL) and saline, respectively. Then the major organs (heart, liver, spleen, lung, kidney, and muscle) were resected after euthanized at 45 min post-injection and followed by imaging. The fluorescence intensity of different organs was compared using the *In Vivo* Xtreme imaging system.

1.10 Ultrasonic/photoacoustic imaging *in vivo*

To track the photoacoustic signal of CA in kidneys, ultrasonic/photoacoustic imaging were performed by the photoacoustic imaging system (Vevo LAZR, Fuji Film Visual Sonics Inc., Japan) and 3D optoacoustic imaging system (LOIS-3D, TomoWave Suzhou Medical Imaging Co.,Ltd, China). Different mice models were i.v. injected with various compounds, and all of the mice were anaesthetized and scanned under

ultrasound and photoacoustic imaging modes at pre-set time points.

1.11 Blood routine analysis

Healthy female F1 mice were randomly divided into two groups (n = 3) and given i.v. injected with saline and **DHU-AKI-3** (500 μL, 0.2 mM) for different times. The blood samples of all the mice were collected into blood collection tubes immediately by enucleation of mouse eyes before being sacrificed and serums isolated for measurements of representative blood parameters (red blood count (RBC), white blood count (WBC), platelets (PLT), and hemoglobin concentration (HGB)) and renal function indexs (blood urea nitrogen (BUN), serum creatinine (sCr), and UA (urine acid)) were then measured by semiautomatic biochemistry analyzer.

1.12 Histopathological studies

Major organs of each group mice were fixed in 4% paraformaldehyde (PFA) immediately following sacrifice, dehydrated in ethanol solution, embedded in paraffin and cut into sections with a thickness of 10 μm for hematoxylin and eosin (H&E) staining. The paraffin was removed by xylene washing and then the sections were incubated with haematoxylin for 4 min and eosin for 2 min, followed by washing with distilled water. The stained sections were examined using a 3DHISTECH Pannoramic 250FLASH scanner.

1.13 Statistical analysis

Quantitative data are presented as mean ± standard deviation (SD). Parametric data were analyzed using Student's t-test. The statistical analysis was conducted using Graph Prism 8.0.1, with $p < 0.05$ considered statistically significant.

1.14 Data availability

The main data supporting the results in this study are available within the paper and Supplementary Information. The raw and analysed datasets generated during the

study are too large to be publicly shared, yet they are available for research purposes from the corresponding author on reasonable request. Source data are provided with this paper.

2 Additional tables

Table S1. Recently published activatable molecular optical probes for diagnosis of diseases.[a]

**Optical probes**	**Imaging modes (*in vivo*)**	**Signal retention time *in vivo***	**Urinalysis modes (*in vitro*)**	**Crosstalk-free multiplex signals**	**Biological application**	**Ref.**
SARS-CyCD	Fluorescence	2 h	Fluorescence	×	SARS-CoV-2 M^{pro}-positive mice	**[4]**
CDIR2	Fluorescence	<2 h	—	×	AKI	**[5]**
CIAKI	Fluorescence and chemiluminescence	~2.5 h	—	×	AKI	**[6]**
MRPs	Fluorescence and chemiluminescence	~2 h	Fluorescence	×	AKI	**[7]**
APNs	Fluorescence	~6 h	Fluorescence	×	Cancer and liver allograft rejection	**[8]**
TAMPs	Fluorescence	~48 h	Fluorescence	×	Tumour-bearing mice	**[9]**
HATS	Fluorescence	4 h	Fluorescence and colorimetric	×	Orthotopic colon cancer	**[10]**
APNso	Fluorescence	4 h	Fluorescence	×	Hepatic ischemia-reperfusion injury mice	**[11]**
ZCRR	Fluorescence	~1 h	Fluorescence	×	Acute renal failure mice	**[12]**
AM Pros	Fluorescence	~3 h	Fluorescence	×	Acute renal allograft rejection	**[13]**
GNP-KTP5-ICG	Fluorescence	>48 h	Fluorescence	×	AKI	**[14]**
**DHU-AKI-3**	**Photoacoustic**	**~60 h**	**Fluorescence and portable colorimetric**	√	**AKI and kidney ischemia reperfusion injury**	**This work**

[a] If no actual data were provided in the references, data were estimated according to the original published figures.

Table S2. The calculated Log P of **DHU-AKI-1, 2, 3**.[a]

Name	**DHU-AKI-1**	**DHU-AKI-2**	**DHU-AKI-3**
cLogP	7.03	1.39	-1.43

[a] The LogP were calculated via XLOPG3 developed by the computational chemical biology group of state key lab of bioorganic chemistry, Shanghai institute of organic chemistry, Chinese academy of science.

3 Additional images and figures

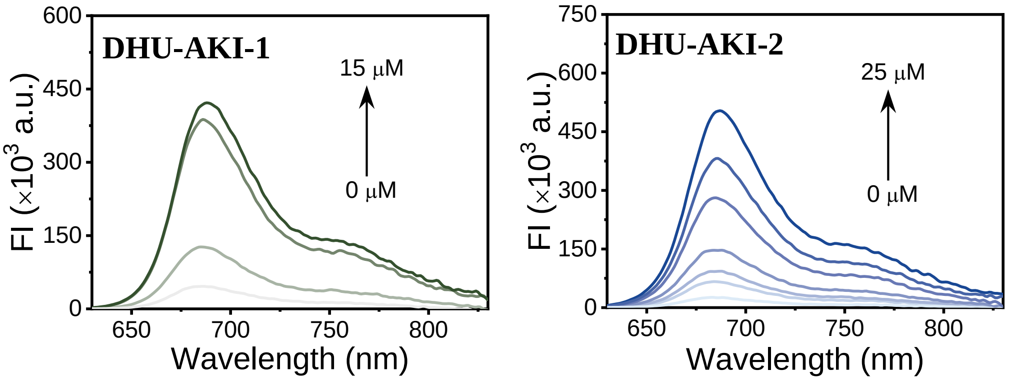

Figure S1. The changes of fluorescence spectra of 10 μM probes (**DHU-AKI-1** and **DHU-AKI-2**) before and after adding various concentration of HOCl in PB (pH 7.4).

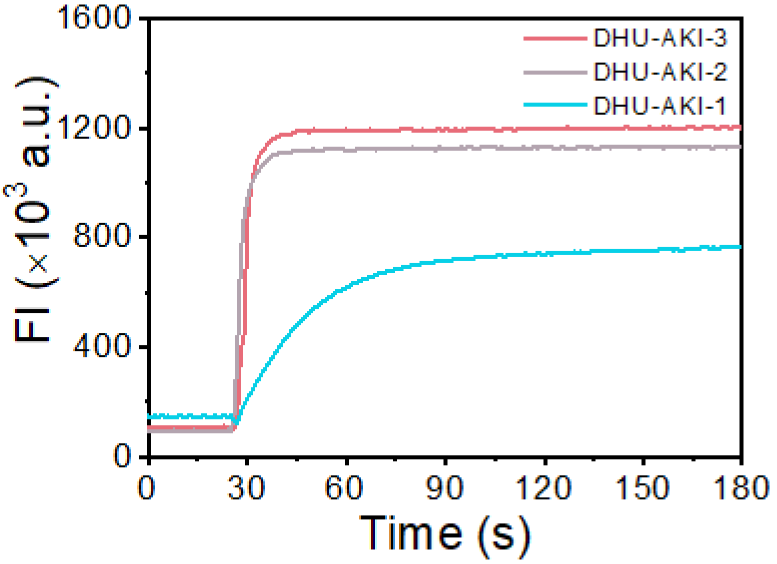

Figure S2. Reaction kinetic profiles of different probes (DHU-AKI-1, DHU-AKI-2, DHU-AKI-3) in the presence of ROS in PB (pH 7.4). Time-dependent changes in the fluorescence intensity of different probes (10 μM) at 685 nm after adding HOCl (25 μM).

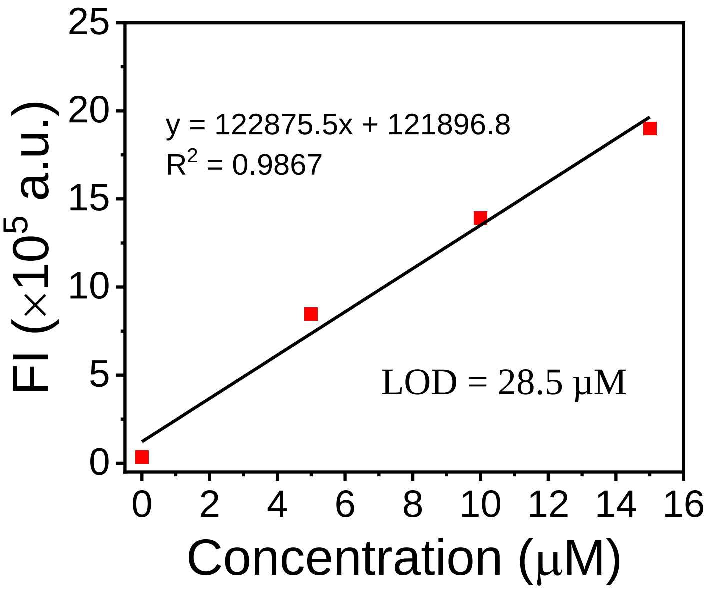

Figure S3. Fluorescence intensity of **DHU-AKI-3** (10 μM) with different concentrations of HOCl (0, 5, 10, and 15 μM) at 686 nm.

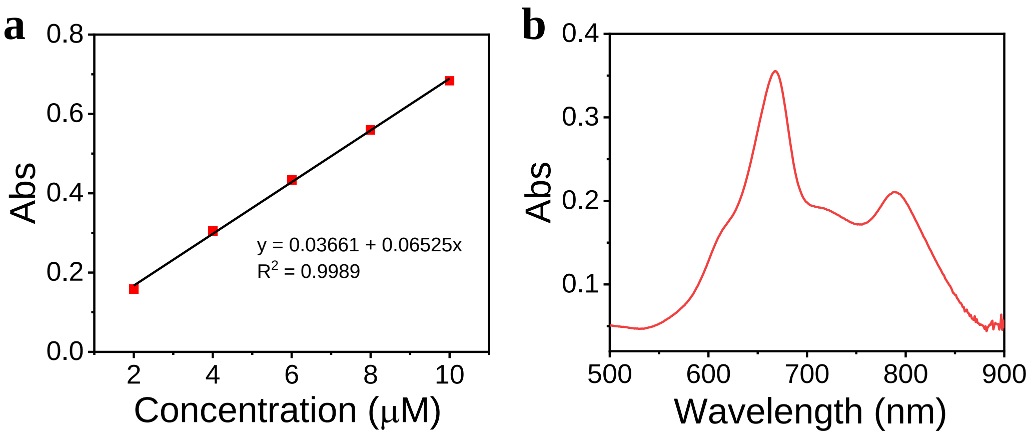

Figure S4. The release efficiency of MB. (a) The linear fit of absorption value (664 nm) versus the concentration of free MB from 2 to 10 μM; (b) the absorption spectra of **DHU-AKI-3** (10 μM in PB) with adding HOCl (30 μM).

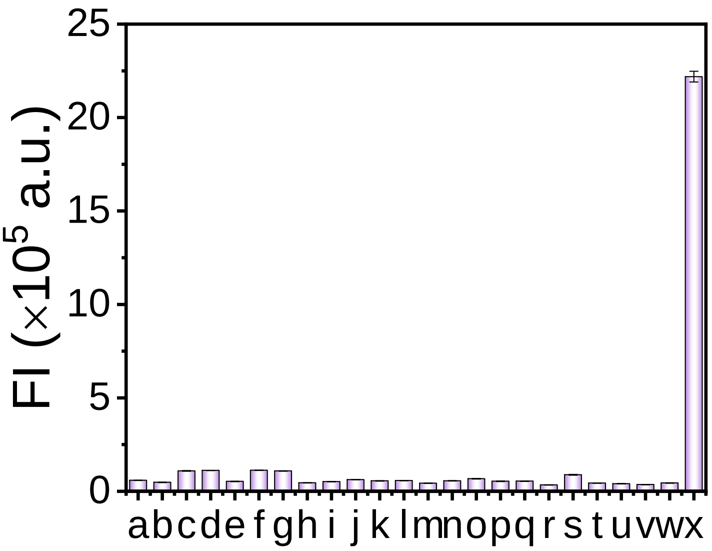

Figure S5. Fluorescence intensity of **DHU-AKI-3** (10 μM) at 686 nm after adding 200 μM various analytes (a) only **DHU-AKI-3**; (b-w) alanine, arginine, asparagine, glutamic acid, glycine, histidine, leucine, lysine, Na_2CO_3, $Na_2S_2O_3$, Na_2SO_4, NaF, NaCl, NaBr, NaI, $NaNO_3$, NaOH, $CaCl_2$, $CuSO_4$, $FeCl_2$, KCl, $MgCl_2$, NH_4Cl; (x) HOCl (20 μM).

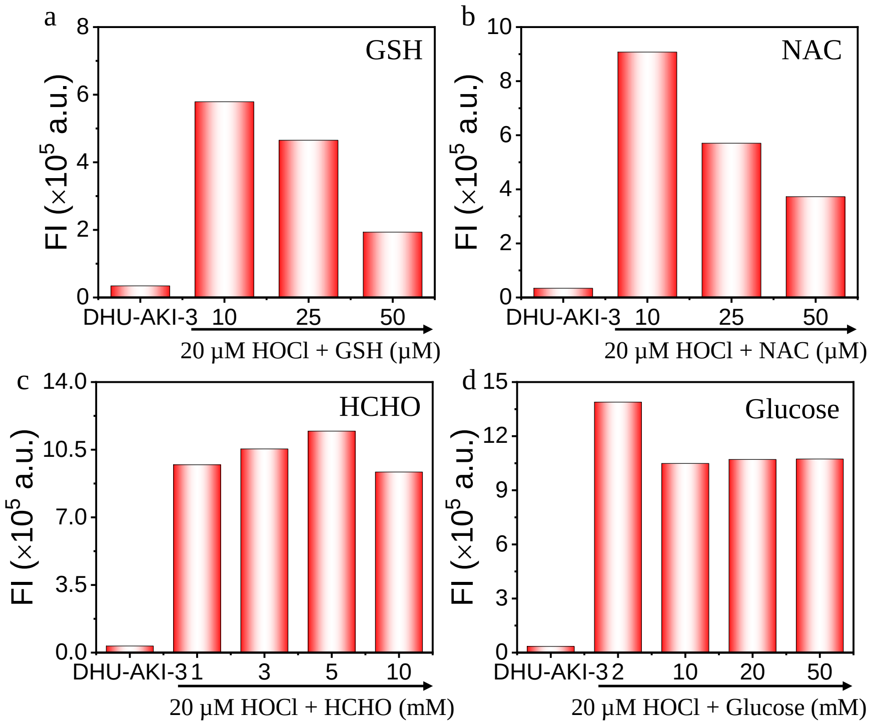

Figure S6. Fluorescence intensity of **DHU-AKI-3** (10 μM) at 686 nm after adding different reducing substances of (a) glutathione (10, 25, 50 μM) and HOCl (20 μM), (b) N-Acetyl-L-cysteine (10, 25, 50 μM) and HOCl (20 μM), (c) formaldehyde (1, 3, 5, 10 mM) and HOCl (20 μM), (d) glucose (2, 10, 20, 50 mM) and HOCl (20 μM).

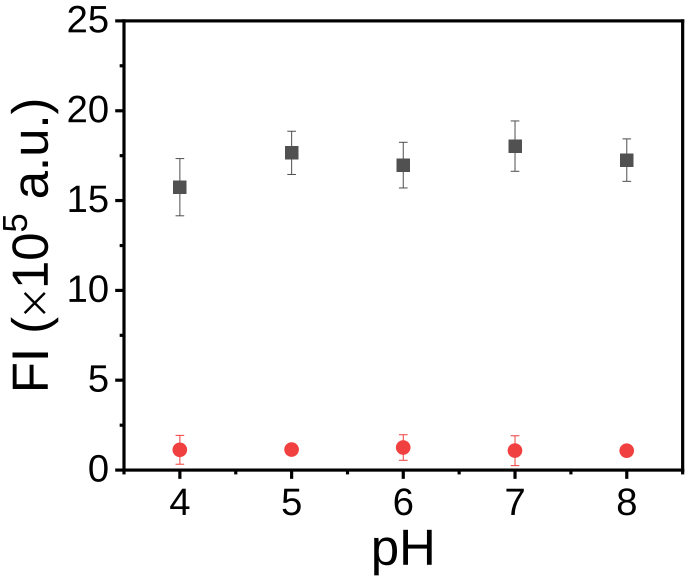

Figure S7. Fluorescence intensity of **DHU-AKI-3** (10 μM) at 686 nm before and after adding HOCl (20 μM) at different pH values, n = 3.

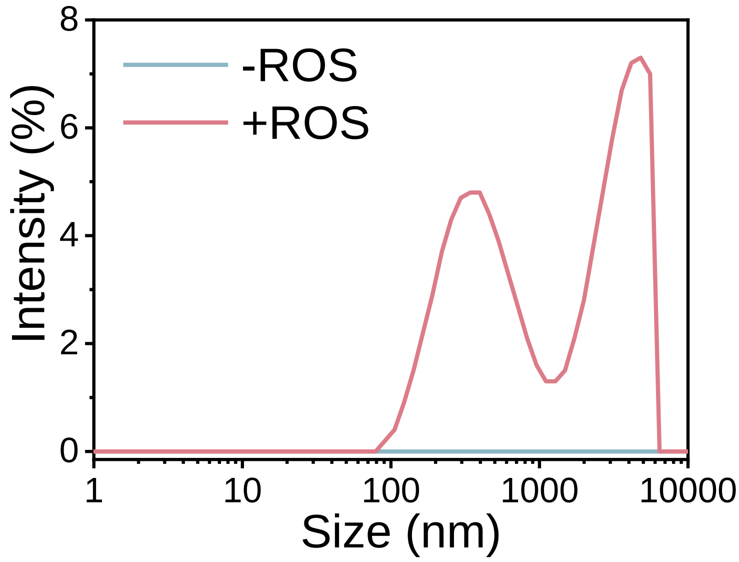

Figure S8. Hydrodynamic diameter of **DHU-AKI-3** (50 μM) without the addition of HOCl and **DHU-AKI-3** (50 μM) in the presence of HOCl (150 μM), respectively.

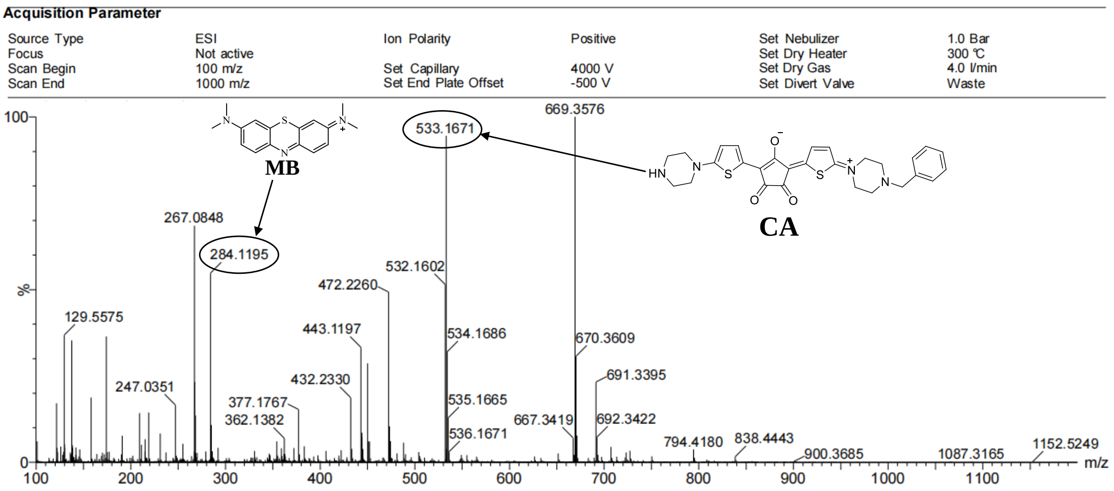

Figure S9. HR MS of **DHU-AKI-3** after incubation with HOCl.

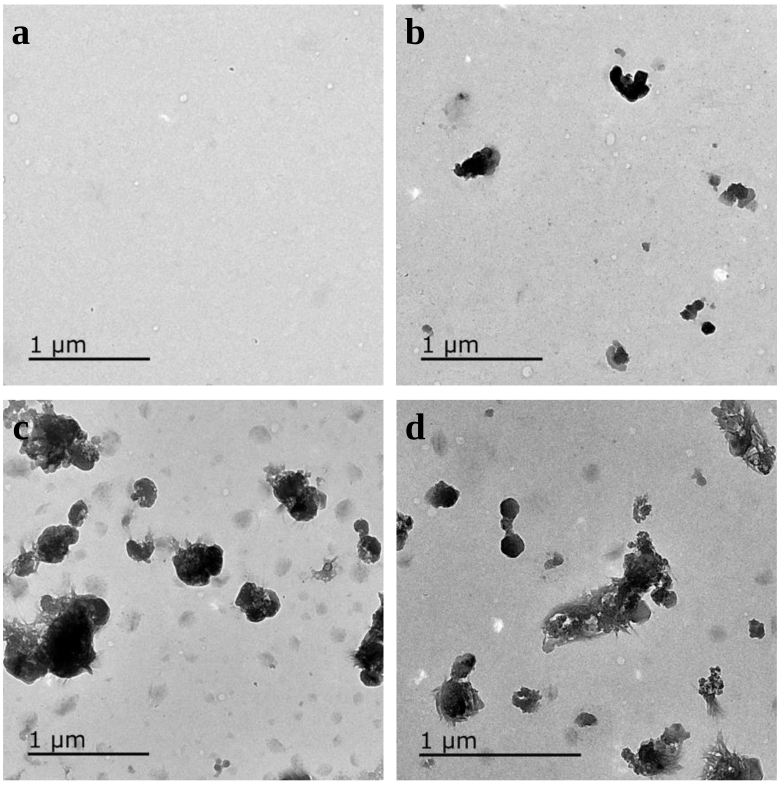

Figure S10. TEM images of different concentrations of **DHU-AKI-3** (a-d: 10, 20, 30, 40 μM) in the presence of HOCl (3 eq).

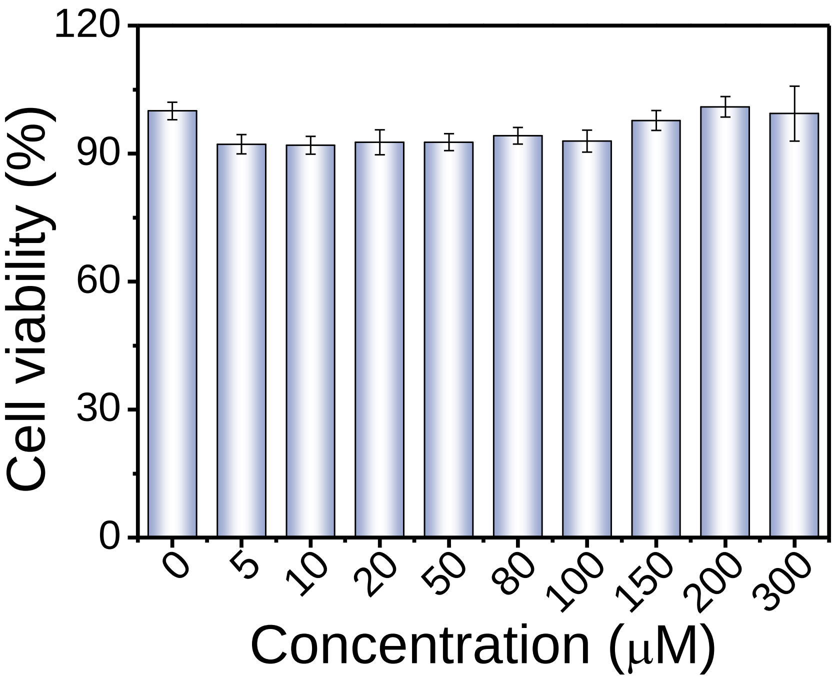

Figure S11. Cell viability. The cell viability of HK-2 cells after incubated with different concentrations of **DHU-AKI-3** (0, 5, 10, 20, 50, 80, 100, 150, 200 and 300 μM) for 24 h measured by CCK-8 assay. Data are presented as mean ± s.d. (n = 6 independent samples).

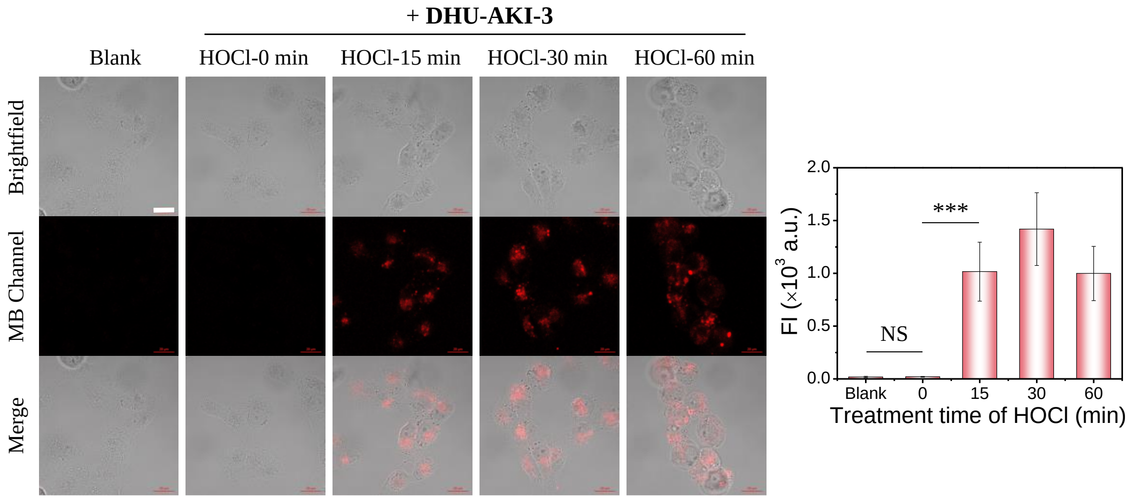

Figure S12. Confocal laser scanning microscope imaging and corresponding quantification. CLSM images of HK-2 cells pre-incubated with 10 μM **DHU-AKI-3** for 3 h before treated with HOCl (50 μM) for different times (0, 15, 30 and 60 min). Excitation wavelength: 633 nm, data are presented as mean ± s.d. (n = 3 independent samples, ***$p < 0.001$, NS: not significant).

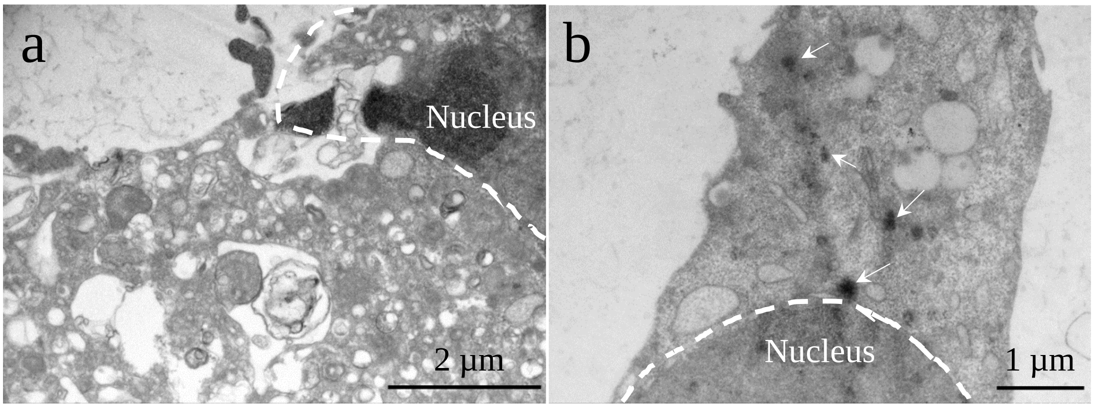

Figure S13. Release of intracellular precipitated CA. Representative TEM images of apoptotic HK-2 cells (pretreated 300 μM cisplatin for 12 h) after incubation of (a) FBS-free culture medium (8 h) and (b) **DHU-AKI-3** (100 μM, 8 h), the white arrows indicate the precipitated particle.

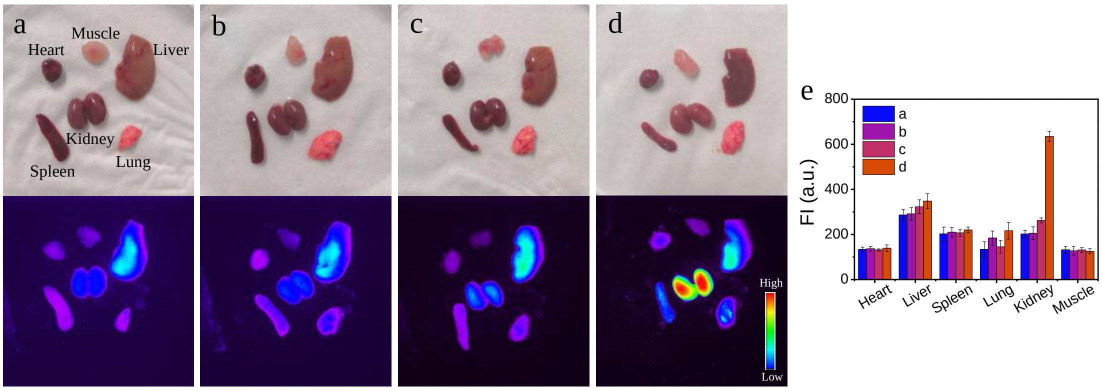

Figure S14. Biodistribution of DHU-AKI-3. Ex vivo fluorescent images of major organs (heart, liver, spleen, lung, kidneys, and tumor) of different mice after treated with saline and **DHU-AKI-3**. (a) healthy mice intravenous (i.v.) injected with saline for 45 min; (b) healthy mice i.v. injected with **DHU-AKI-3** for 45 min; (c) AKI mice i.v. injected with saline for 45 min; (d) AKI mice i.v. injected with **DHU-AKI-3** for 45 min. (e) Quantification of average fluorescence intensity of major organs in groups a-d.

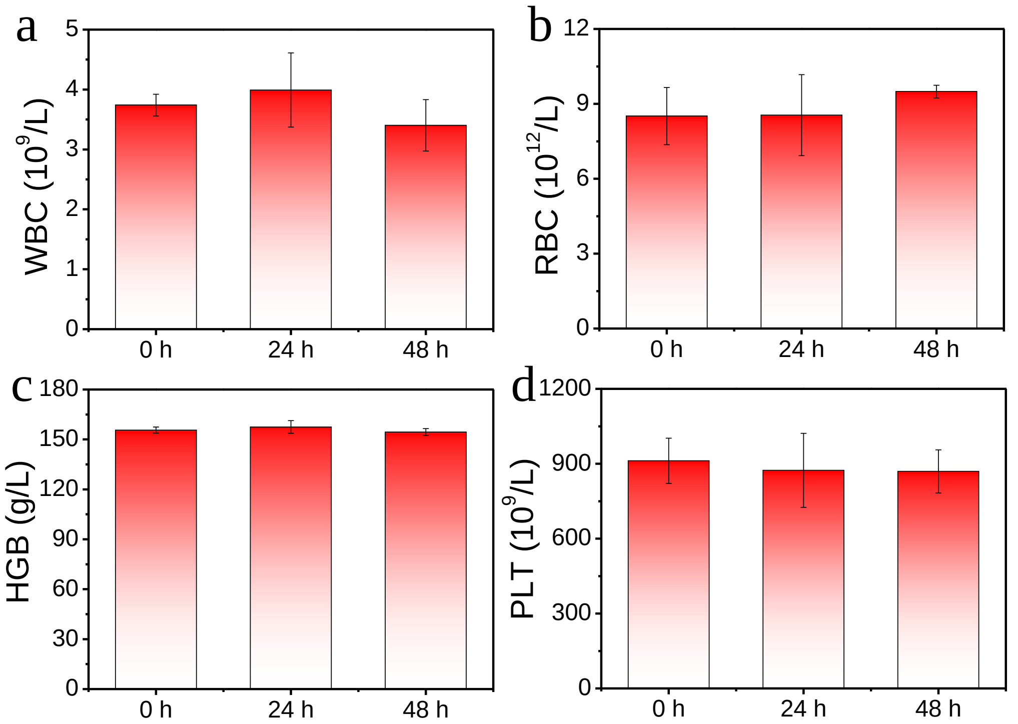

Figure S15. Biosecurity evaluation of DHU-AKI-3. Blood routine analysis of healthy mice after i.v. injected with **DHU-AKI-3** (10 mg/kg, 200 μL) for different times (0, 24, and 48 h). These data are the mean ± s.d.; $n = 3$ independent experiments for (a) white blood count (WBC); (b) red blood count (RBC); (c) hemoglobin concentration (HGB); (d) platelets (PLT).

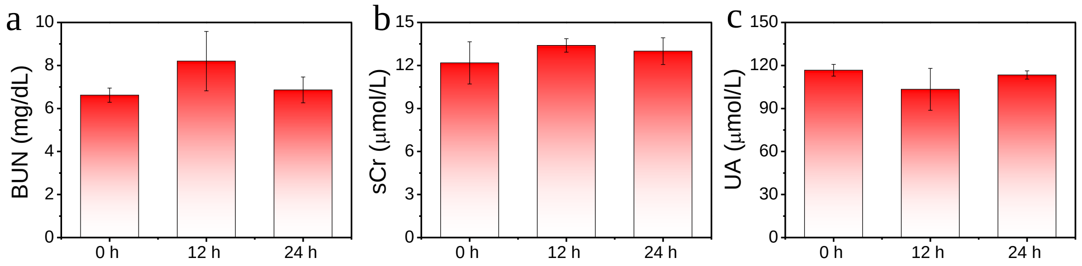

Figure S16. Biochemical analysis of renal function in healthy mice after i.v. injected with DHU-AKI-3 (10 mg/kg, 200 μL) for different times (0, 12, and 24 h). These data are the mean ± s.d.; $n = 3$ independent experiments for (a) blood urea nitrogen (BUN); (b) serum creatinine (SCr); (c) uric acid (UA).

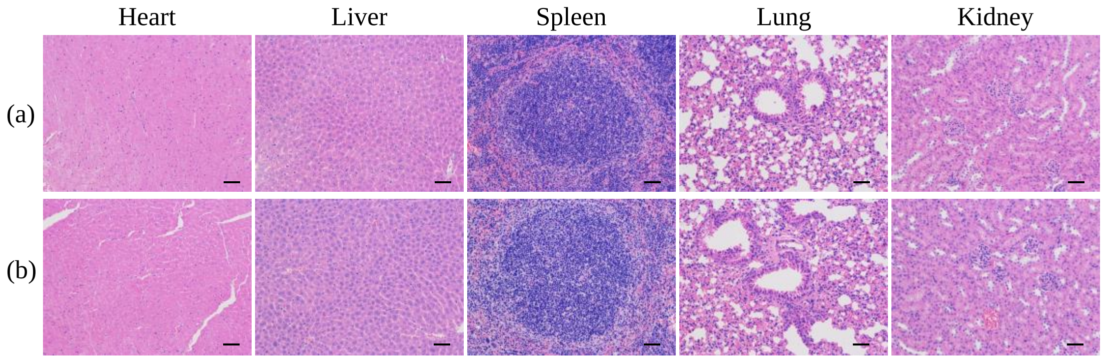

Figure S17. Histological analysis. H&E staining images of major organs (heart, liver, spleen, lung, kidney) of healthy mice after i.v. injected with (a) saline, (b) **DHU-AKI-3** (10 mg/kg, 200 μL) for 48 h, scale bars = 50 μm.

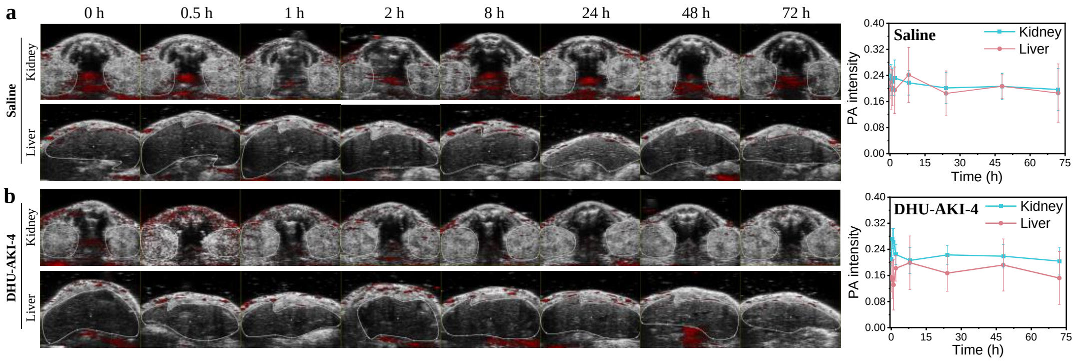

Figure S18. (a-b) The photoacoustic imaging of AKI mice in kidneys and liver after i.v. injected with saline and DHU-AKI-4 (10 mg/kg, 200 μL) at different post-injection time points (0, 0.5, 1, 2, 8, 24, 48 and 72 h). The graph on the right showed the corresponding photoacoustic signal intensity.

4 NMR and HR-MS spectra

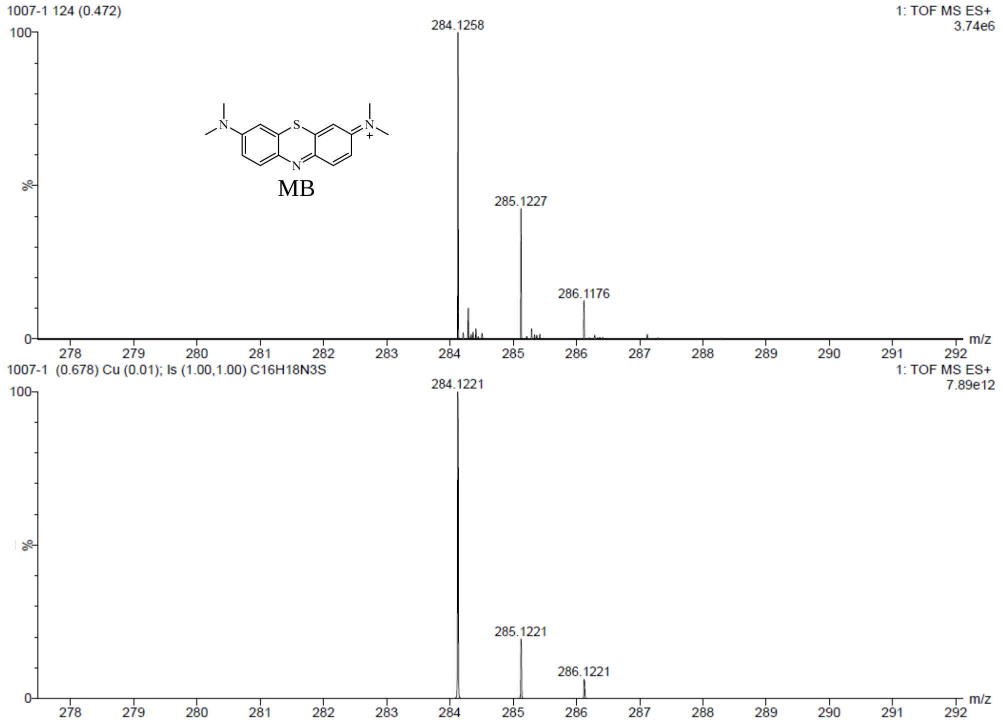

Figure S19. HR MS of AKI mouse urine sample after injection of **DHU-AKI-3.**

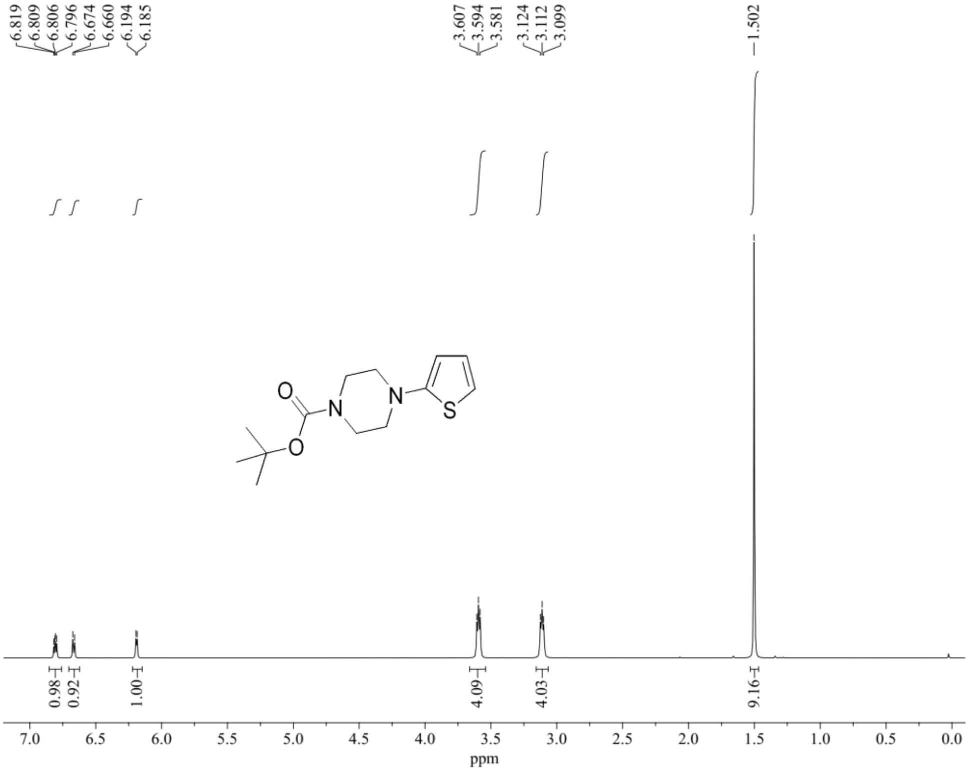

Figure S20. ^{1}H NMR of compound **3** in $CDCl_3$.

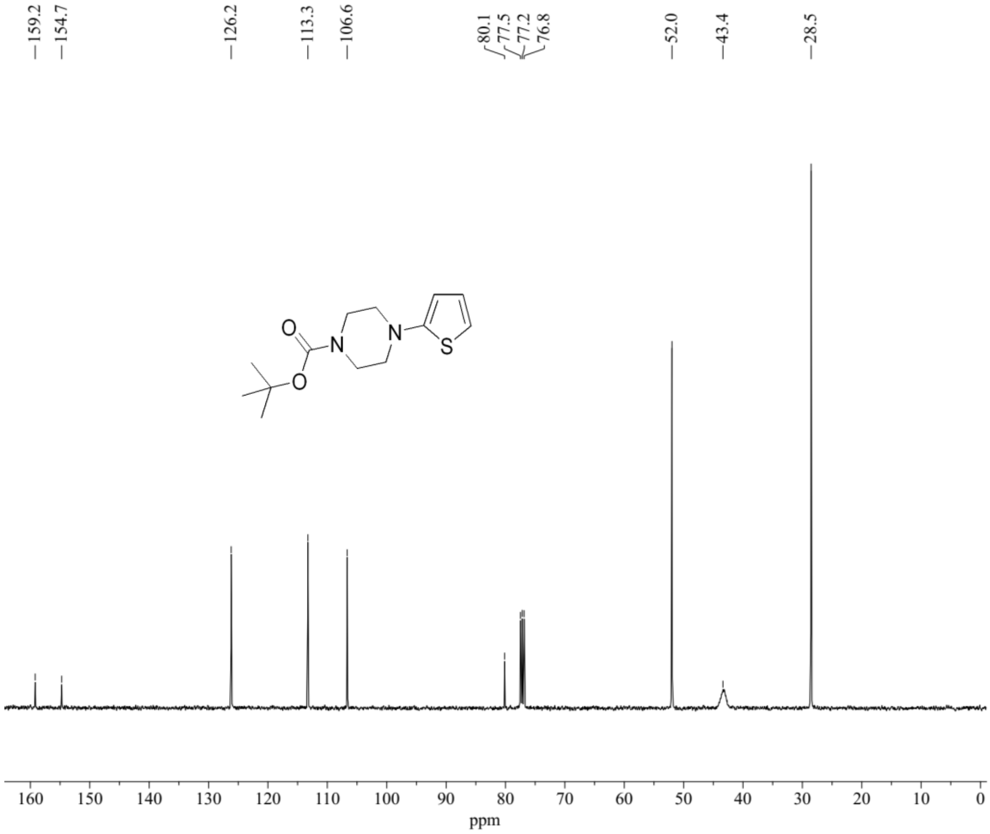

Figure S21. ^{13}C NMR of compound **3** in $CDCl_3$.

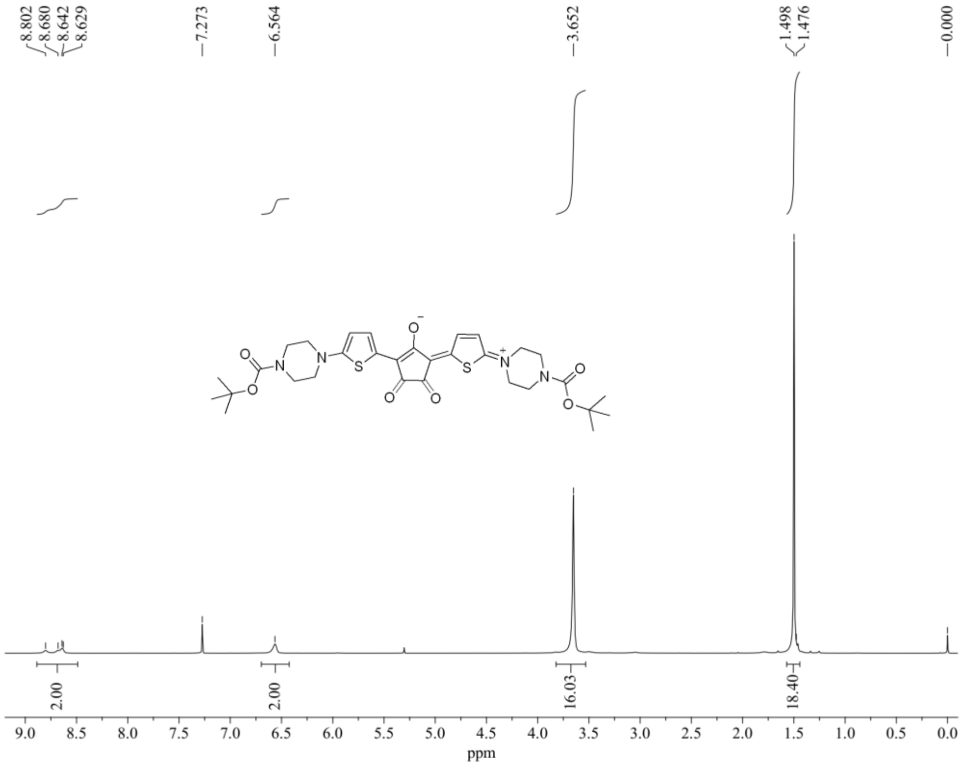

Figure S22. ^{1}H NMR of compound **5** in $CDCl_3$.

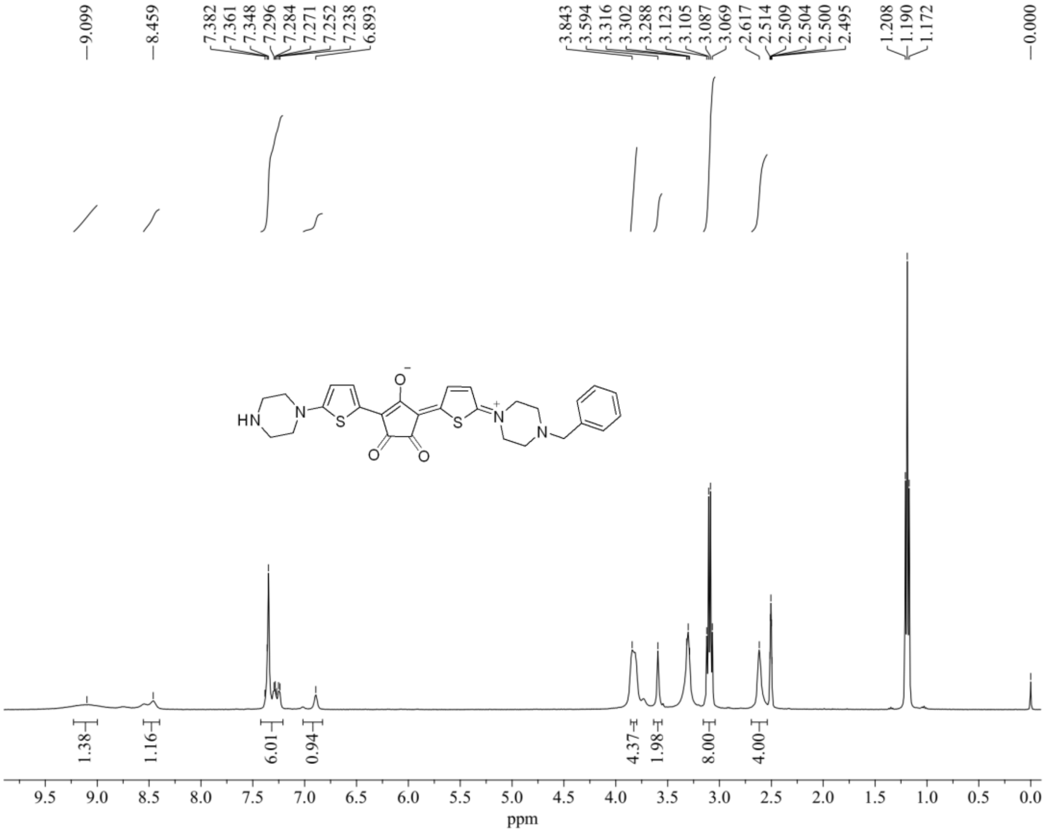

Figure S23. ^{1}H NMR of **CA** in DMSO-d_6.

Acquisition Parameter

Source Type	ESI	Ion Polarity	Positive	Set Nebulizer	1.0 Bar
Focus	Not active			Set Dry Heater	250 ℃
Scan Begin	100 m/z	Set Capillary	4000 V	Set Dry Gas	4.0 l/min
Scan End	1000 m/z	Set End Plate Offset	-500 V	Set Divert Valve	Waste

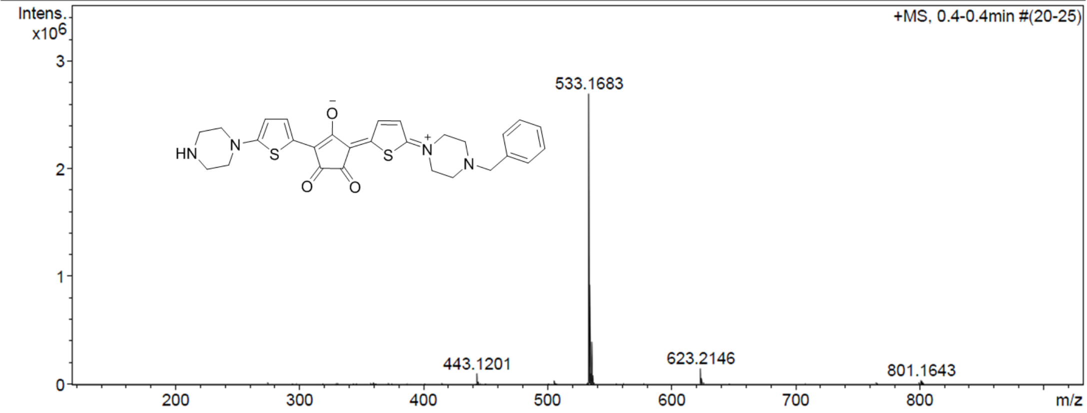

Figure S24. HR MS of **CA.**

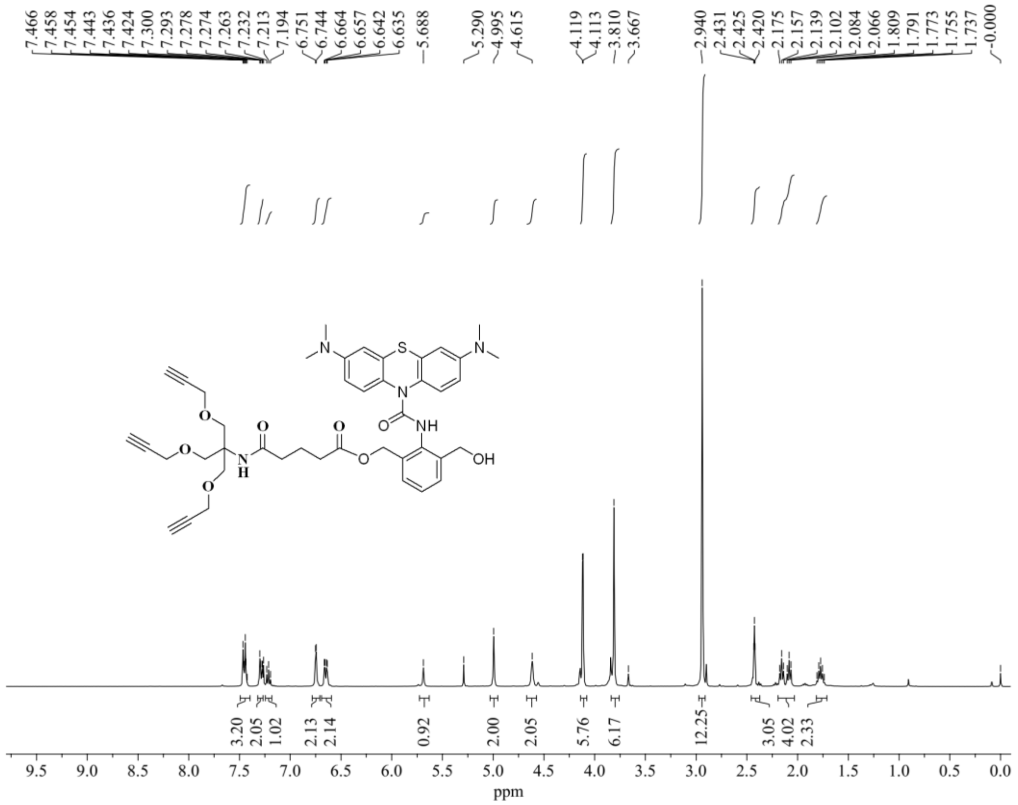

Figure S25. ^{1}H NMR of compound **11** in $CDCl_3$.

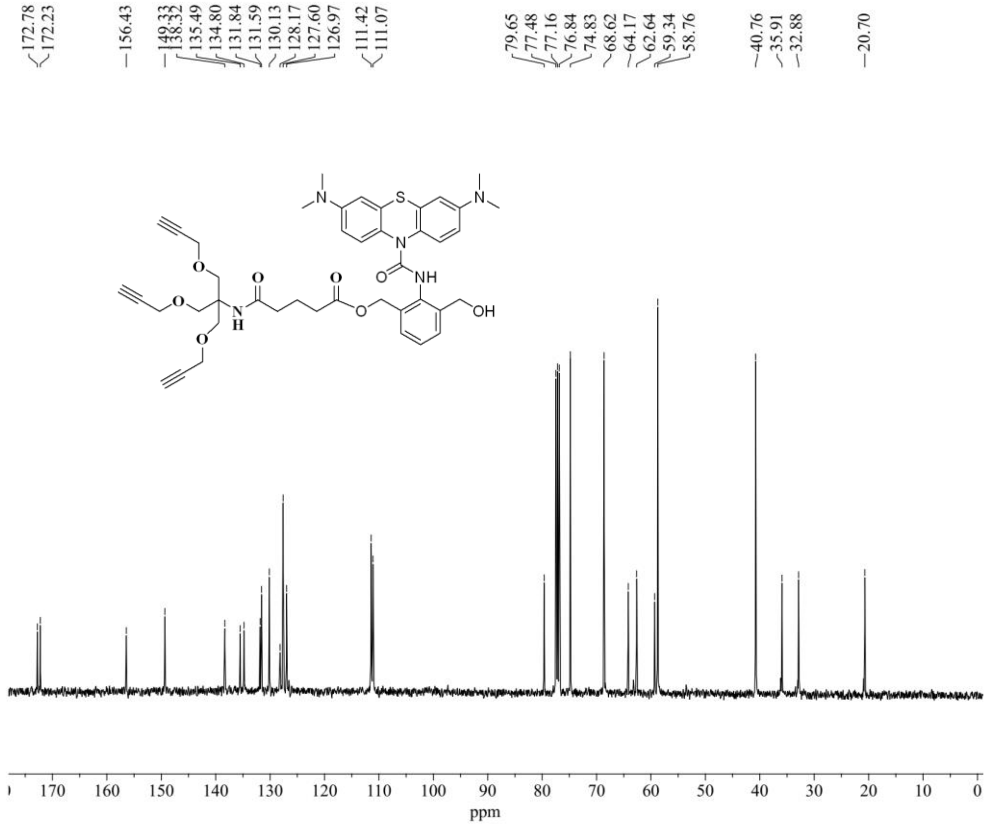

Figure S26. ^{13}C NMR of compound **11** in $CDCl_3$.

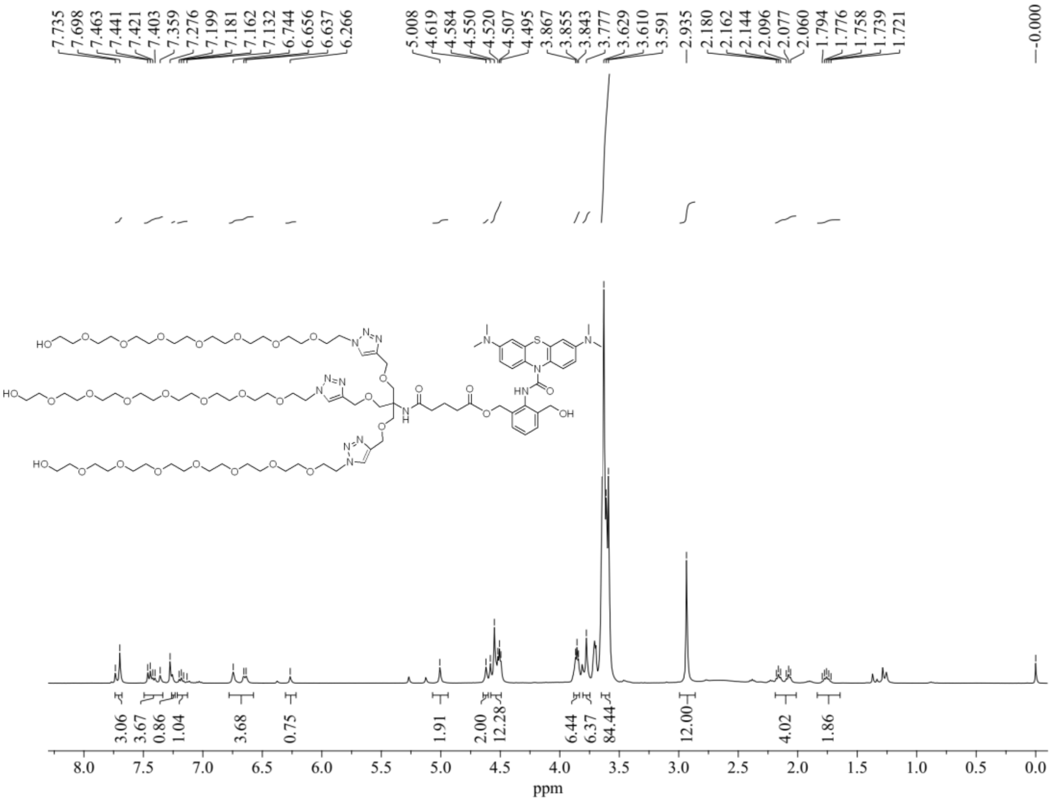

Figure S27. ^{1}H NMR of compound **DHU-AKI-4** in $CDCl_3$.

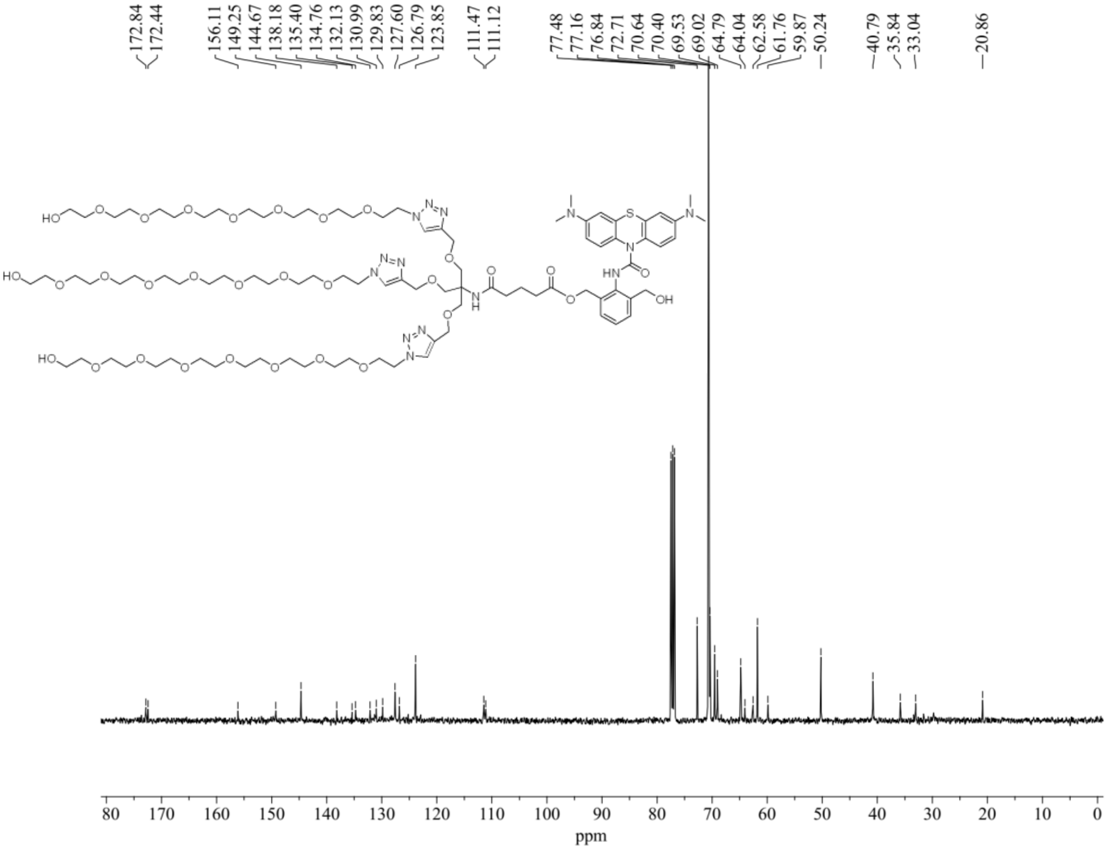

Figure S28. ^{13}C NMR of **DHU-AKI-4** in $CDCl_3$.

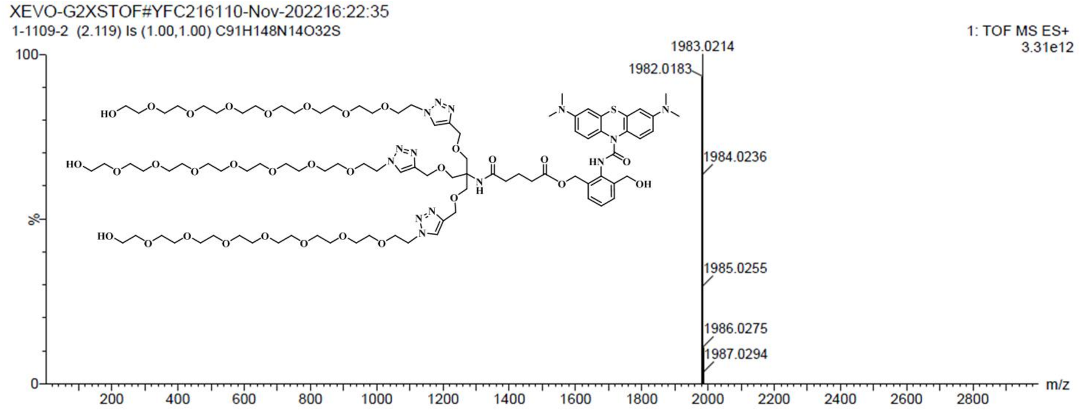

Figure S29. HR MS of **DHU-AKI-4.**

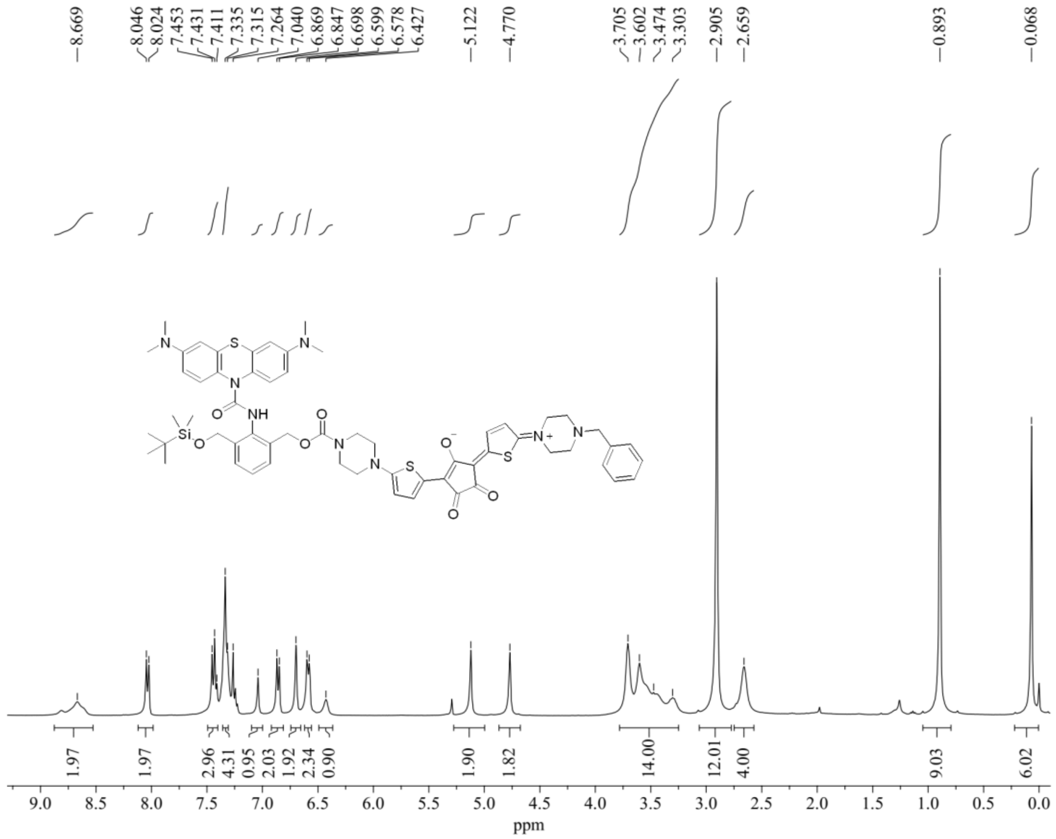

Figure S30. ^{1}H NMR of compound **15** in $CDCl_3$.

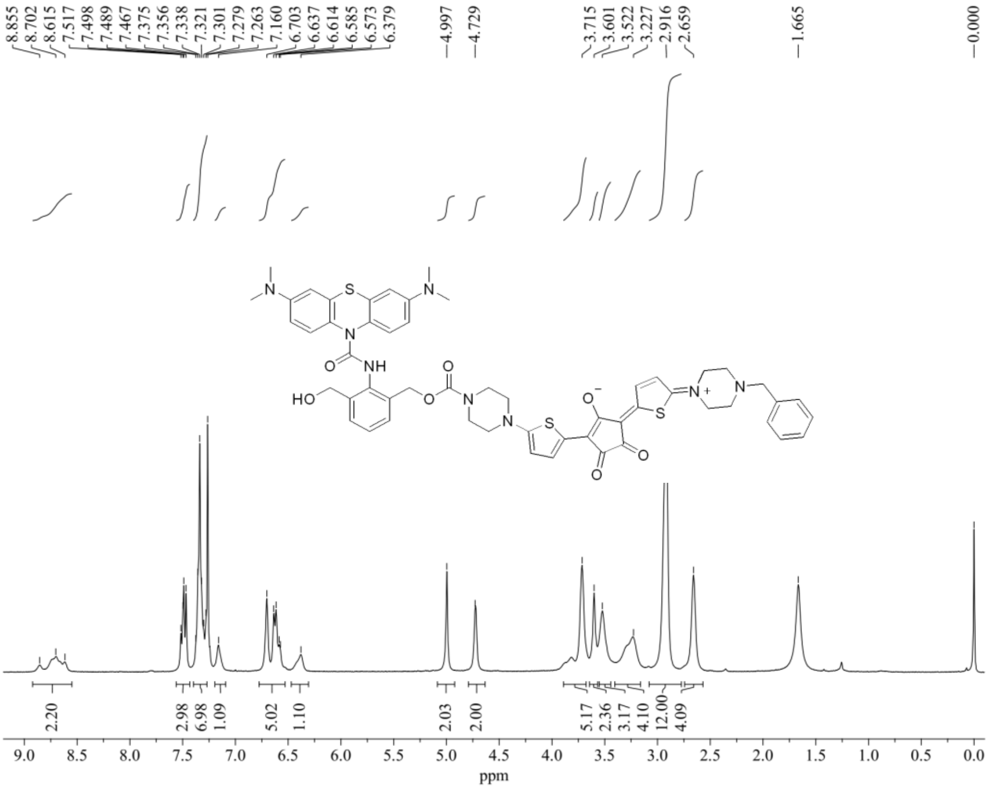

Figure S31. ^{1}H NMR of **DHU-AKI-1** in $CDCl_3$.

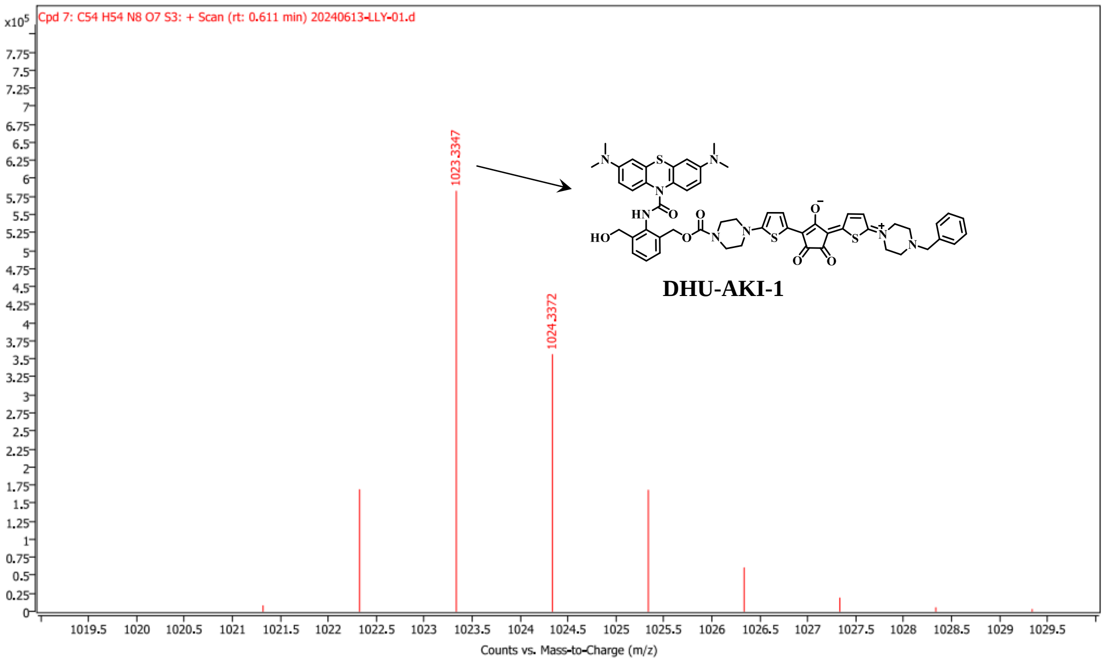

Figure S32. HR MS of **DHU-AKI-1.**

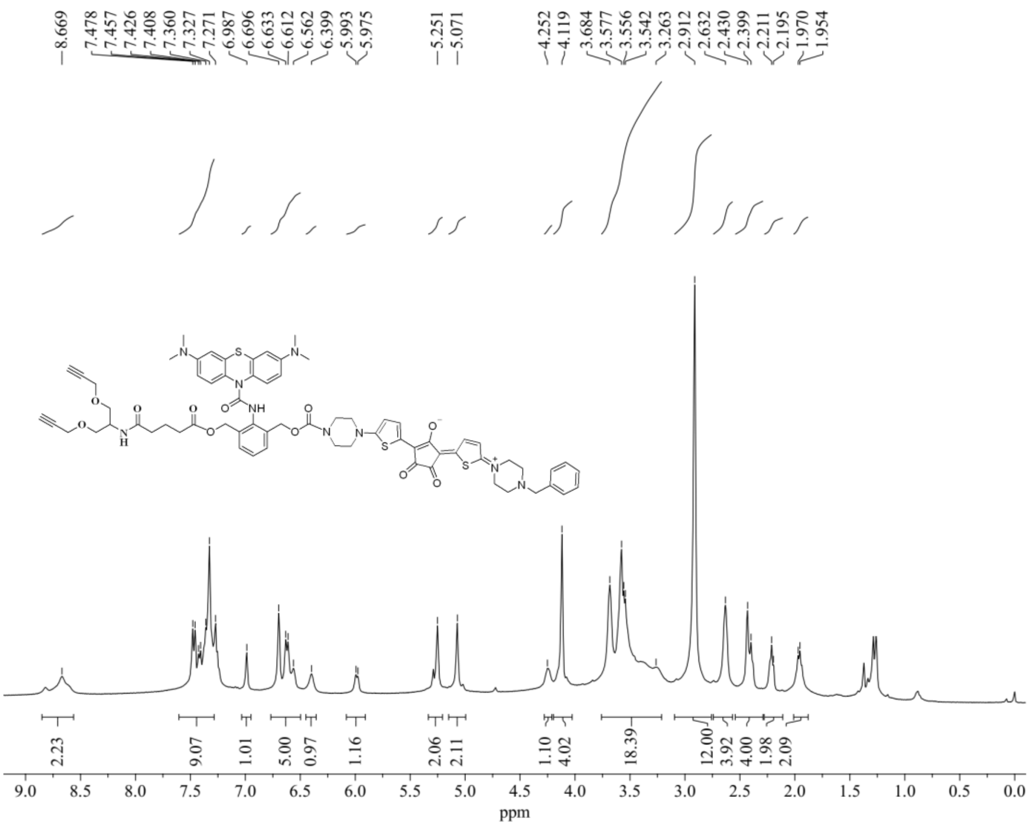

Figure S33. ^{1}H NMR of compound **17** in $CDCl_3$.

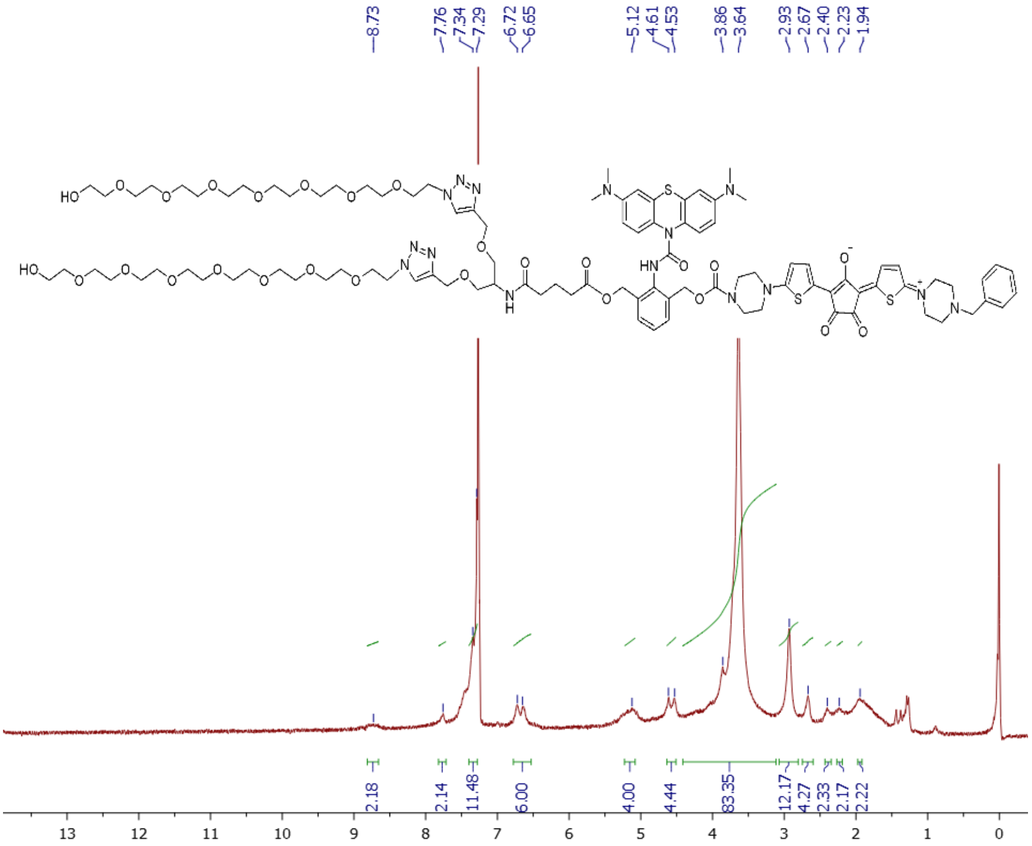

Figure S34. ^{1}H NMR of **DHU-AKI-2** in $CDCl_3$.

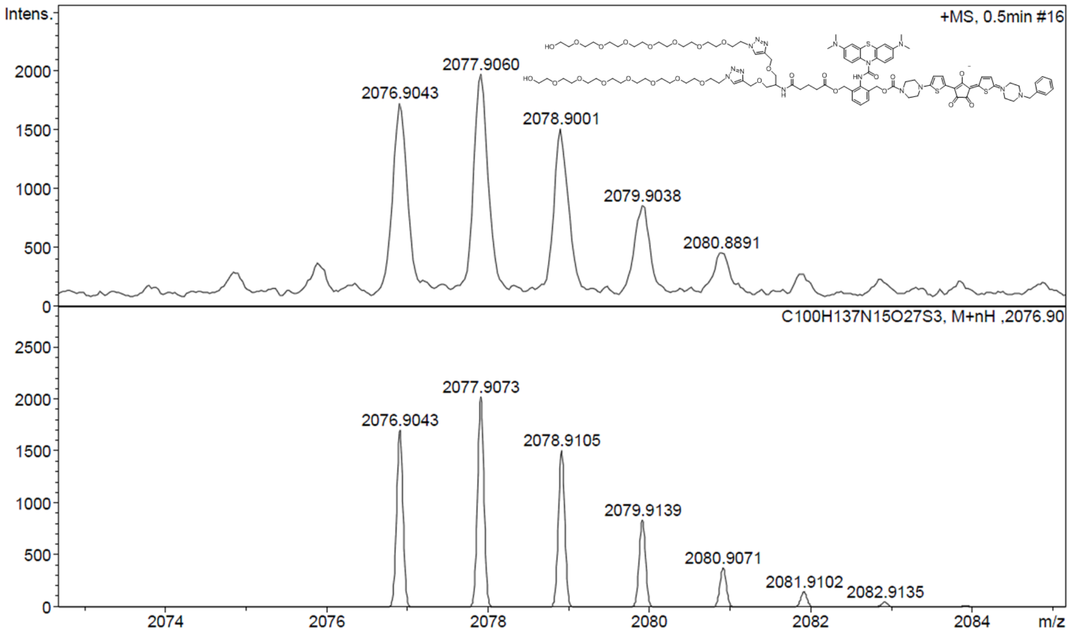

Figure S35. HR MS of **DHU-AKI-2.**

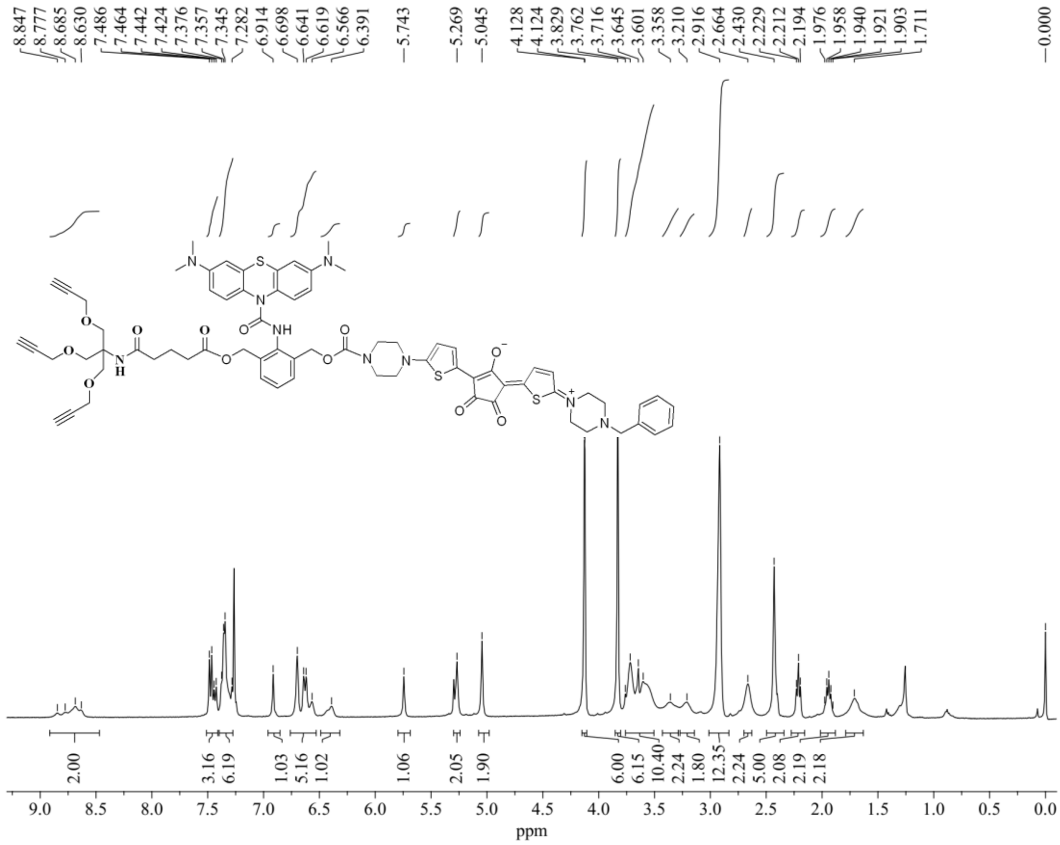

Figure S36. ^{1}H NMR of compound **18** in $CDCl_3$.

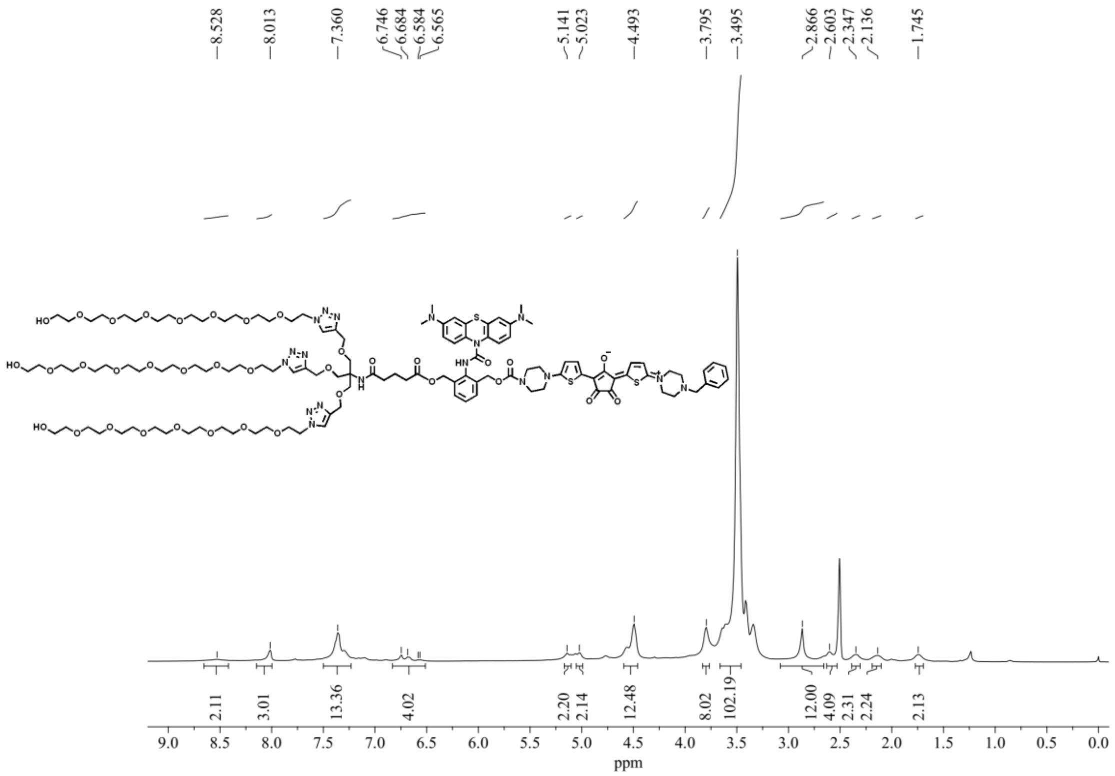

Figure S37. ^{1}H NMR of **DHU-AKI-3** in DMSO-d_6.

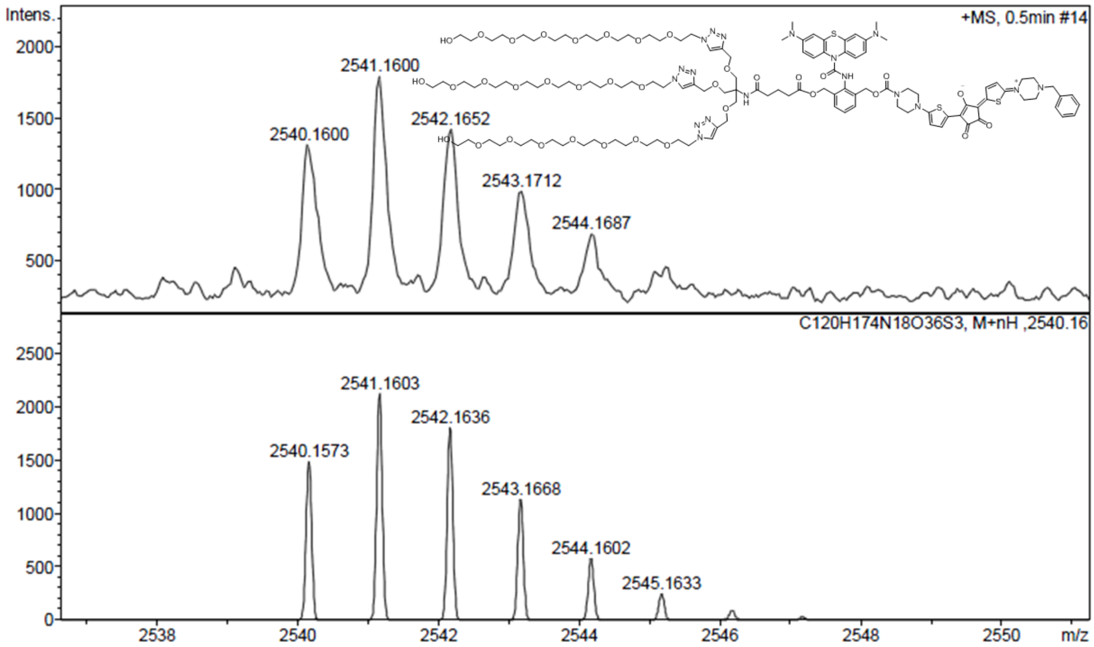

Figure S38. HR MS of **DHU-AKI-3.**

5 References

1. Liu F, Liu L, Liu D, et al. An excipient-free “sugar-coated bullet” for the targeted treatment of orthotopic hepatocellular carcinoma. *Chem Sci* 2022; **13**: 10815–10823.

2. Liu L, Liu F, Liu D, et al. A smart theranostic prodrug system activated by reactive oxygen species for regional chemotherapy of metastatic cancer. *Angew Chem Int Ed* 2022; **134**: e202116807.

3. Liu L, Jiang L, Yuan W, et al. Dual-modality detection of early-stage drug-induced acute kidney injury by an activatable probe. *ACS Sens* 2020; **5**: 2457–2466.

4. Liew SS, Zeng Z, Cheng P, et al. Renal-clearable molecular probe for near-infrared fluorescence imaging and urinalysis of SARS-CoV-2. *J Am Chem Soc* 2021; **143**: 18827–18831.

5. Huang J, Xie C, Zhang X, et al. Renal-clearable molecular semiconductor for second near-infrared fluorescence imaging of kidney dysfunction. *Angew Chem Int Ed* 2019; **58**: 15120–15127.

6. Huang J, Lyu Y, Li J, et al. A Renal-Clearable Duplex Optical Reporter for Real-Time Imaging of Contrast-Induced Acute Kidney Injury. *Angew Chem Int Ed* 2019; **131**: 17960–17968.

7. Huang J, Li J, Lyu Y, et al. Molecular optical imaging probes for early diagnosis of drug-induced acute kidney injury. *Nat Mater* 2019; **18**: 1133–1143.

8. Huang J, Chen X, Jiang Y, et al. Renal clearable polyfluorophore nanosensors for early diagnosis of cancer and allograft rejection. *Nat Mater* 2022; **21**: 598–607.

9. He S, Cheng P, Pu K. Activatable near-infrared probes for the detection of specific populations of tumour-infiltrating leukocytes in vivo and in urine. *Nat Biomed Eng* 2023; **7**: 281–297.

10. Xu C, Xu M, Hu Y, et al. Ingestible Artificial Urinary Biomarker Probes for Urine Test of Gastrointestinal Cancer. *Adv Mater* 2024; **36**: 2314084.

11. Huang J, Xian S, Liu Y, et al. A Renally Clearable Activatable Polymeric Nanoprobe for Early Detection of Hepatic Ischemia-Reperfusion Injury. *Adv Mater* 2022; **34**: 2201357.

12. Zhou Y, Zhu L, Liu B, et al. Tailored Zwitterionic Hemicyanine Reporters for Early Diagnosis and Prognostic Assessment of Acute Renal Failure. *Angew Chem Int Ed* 2023; **62**: e202315457.

13. Cheng P, Wang R, He S, et al. Artificial urinary biomarkers for early diagnosis of acute renal allograft rejection. *Angew Chem Int Ed* 2023; **62**: e202306539.

14. Chen Y, Pei P, Lei Z, et al. A promising NIR-II fluorescent sensor for peptide-mediated long-term monitoring of kidney dysfunction. *Angew Chem Int Ed* 2021; **133**: 15943–15949.
